# Supplementary material for: Endoglycosidase assay using enzymatically synthesized fluorophore-labeled glycans as substrates to uncover enzyme substrate specificities
Source: Commun Biol. 2022 May 25;5:501. doi: 10.1038/s42003-022-03444-3 (PMC9132957; doi:10.1038/s42003-022-03444-3)
Supplement: Supplementary file 2 — Supplementary Information [file 42003_2022_3444_MOESM2_ESM.pdf]

Endoglycosidase Assay Using Enzymatically Synthesized Fluorophore-labeled Glycans as Substrates to Uncover Enzyme Substrate Specificities

Key words: Glycosyltransferase/Endo-β-Gal/EndoS/EndoS2/N-glycans/Polylactosamine/Enzymatic synthesis

Running title: Endoglycosidase assay

Zhengliang L Wu[1*], James M Ertelt[1]

[1]Bio-techne, R&D Systems, Inc. 614 McKinley Place N.E.
Minneapolis, MN, 55413, USA

*Correspondence: Phone: 612-656-4544. Email: leon.wu@bio-techne.com,

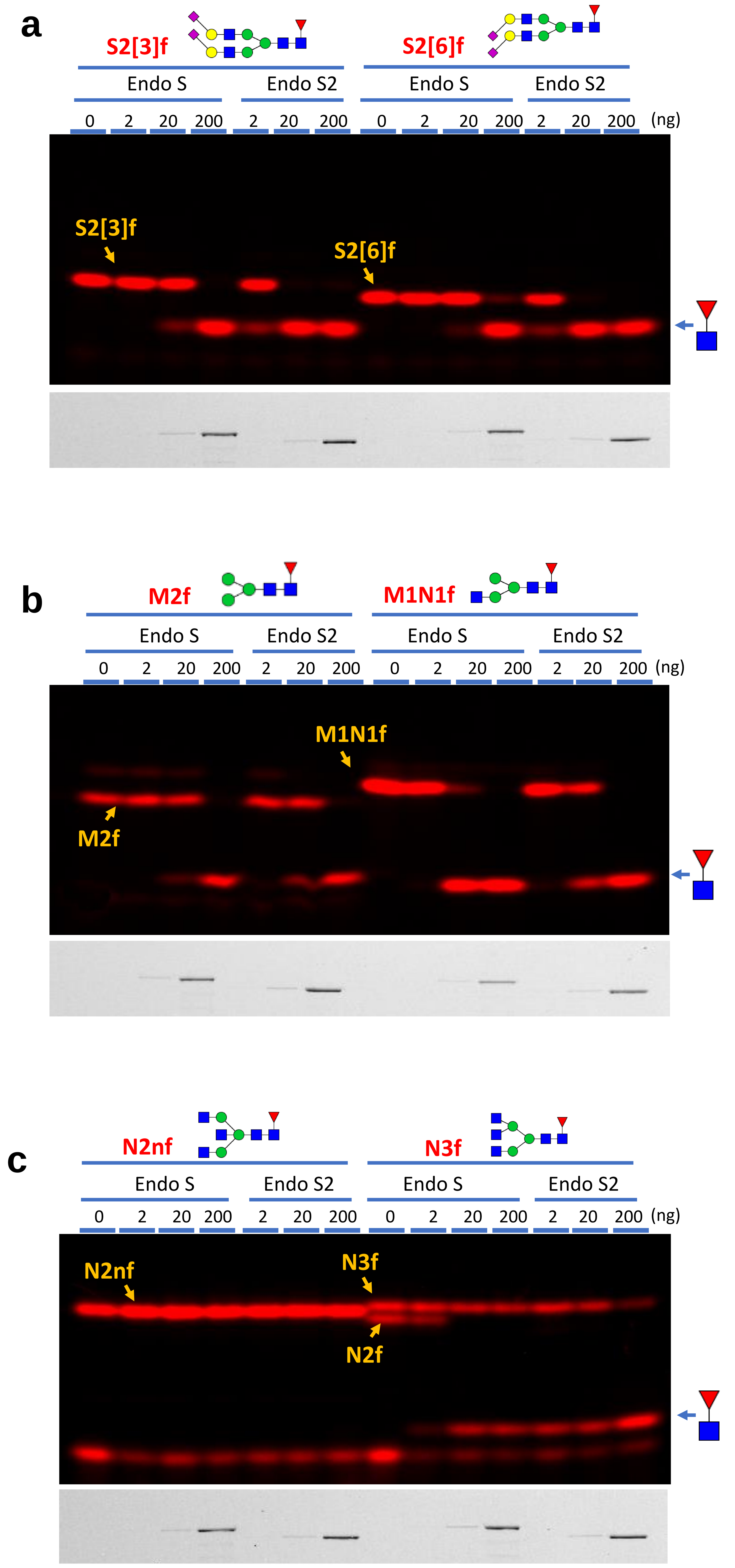

Supplemental Fig. 1. Thorough digestion of different glycans with indicated amounts of Endo S/S2. All digestions were performed at three different amounts of Endo S/S2 and were incubated at 37° C for 18 hours. **a** Digestion on S2[3]f and S2[6]f. **b** Digestion on Group II glycans. **c** Digestions on Group III glycans.

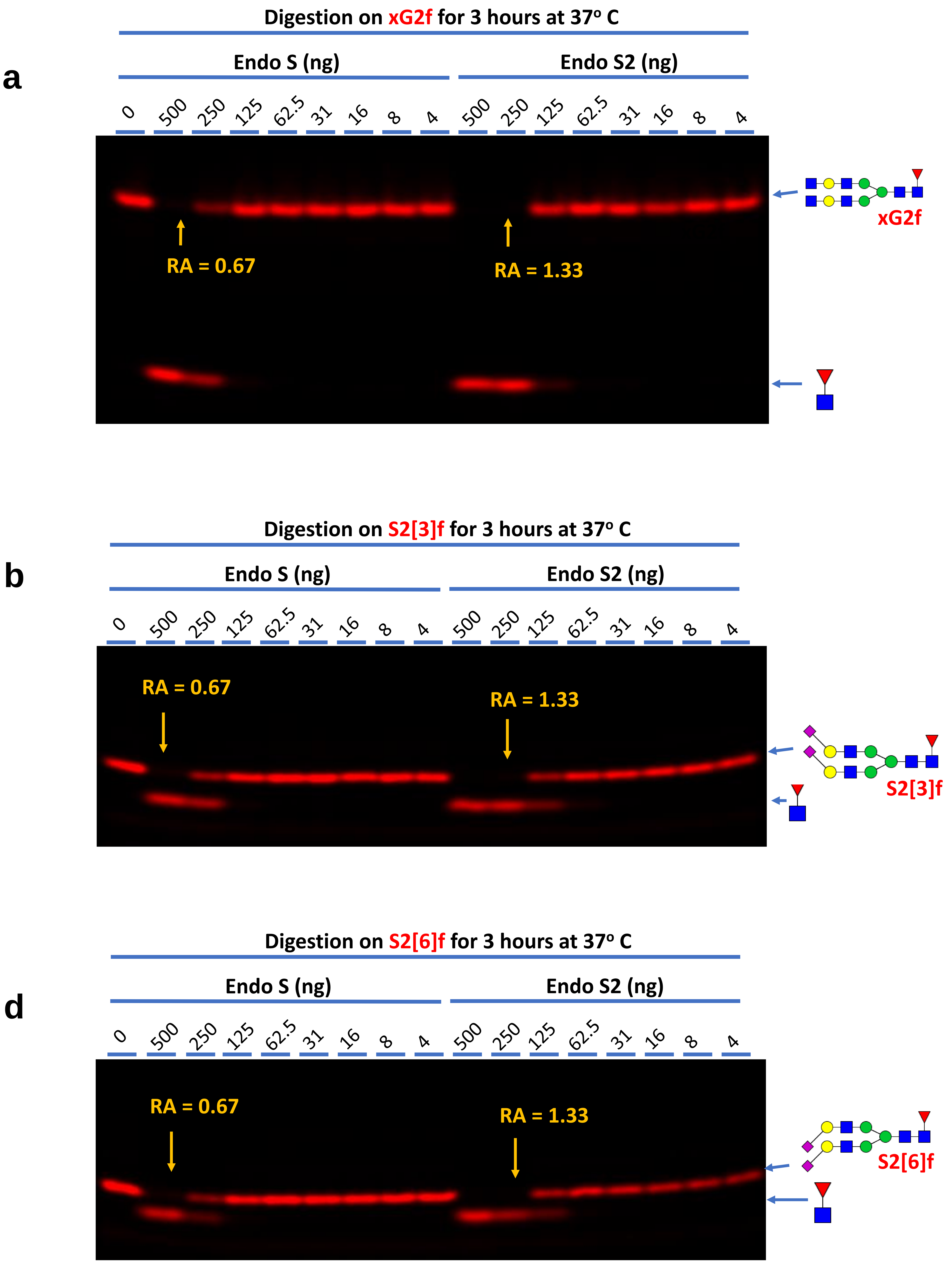

Supplemental Figure 2. Relative activity of Endo S/S2 on xG2f, S2[3]f and S2[6]f.
All digestions were performed with the indicated amounts of Endo S or Endo S2 at 37º C for 3 hours and separated on 15% gels. For simplicity, only the images for glycans are shown. The data suggests that chain elongation reduces the activity of both enzymes and Endo S2 is consistently more active than Endo S. **a** Assay on xG2f. **b** Assay on S2[3]f. **c** Assay on S2[6]f.

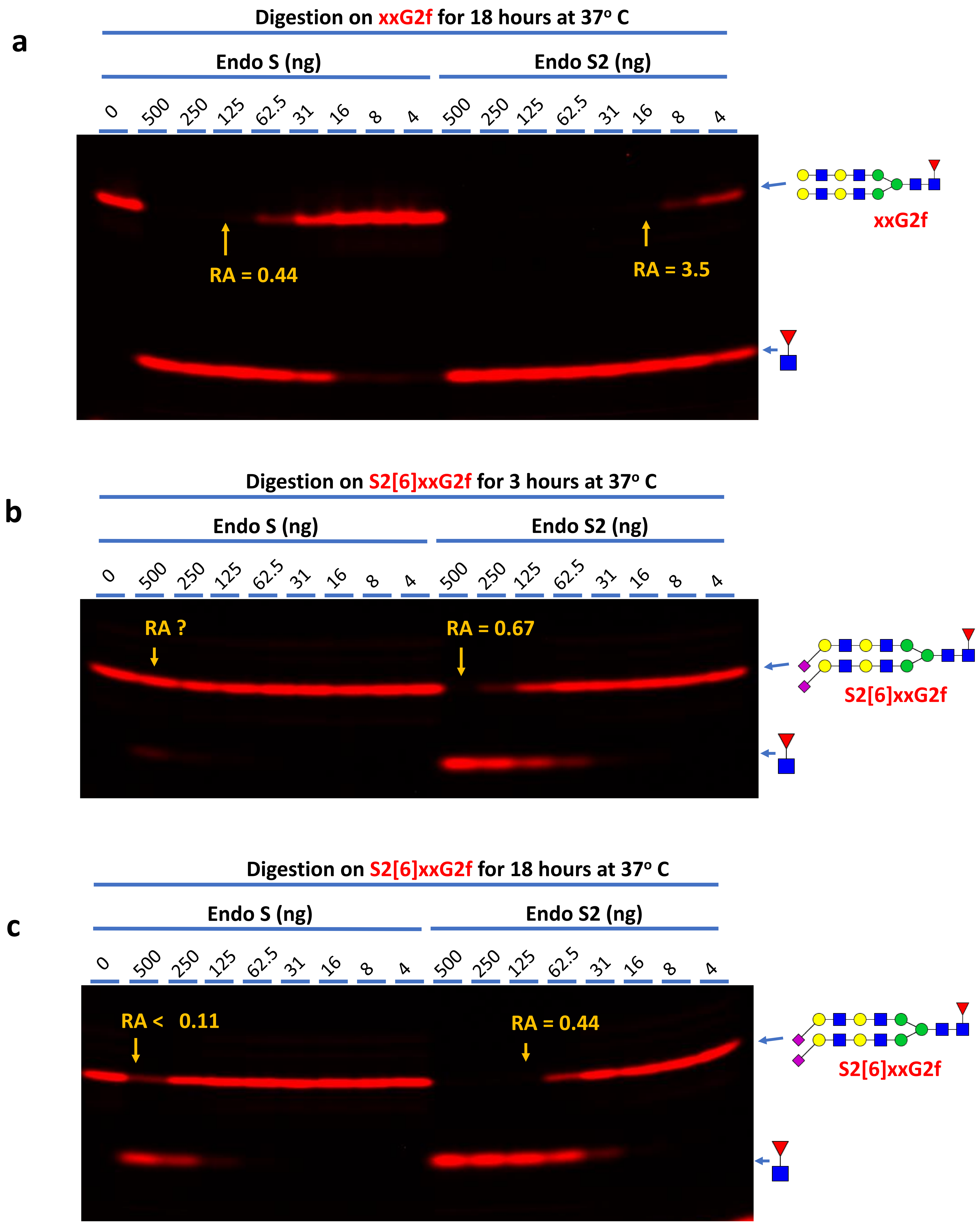

Supplemental Figure 3. Relative activity of Endo S/S2 on xxGf and S2[6]xxG2f.
All digestions were performed with the indicated amounts of Endo S or Endo S2 at 37° C and separated on 15% gels. For simplicity, only the images for glycans are shown. Comparing to the activity of both enzymes on N2f in Figure 3, the results again demonstrate that chain elongation reduces the activity of both enzymes and Endo S2 is consistently more active than Endo S. **a** Assay on xxG2f for 18 hours. **b** Assay on S2[6]xxG2f for 3 hours. **c** Assay on S2[6]xxG2f for 18 hours. Since that none of the dose of Endo S achieved 95% cleavage on S2[6]xxG2f in **b**, the experiment was repeated in **c** with incubation time increased to 18 hours.

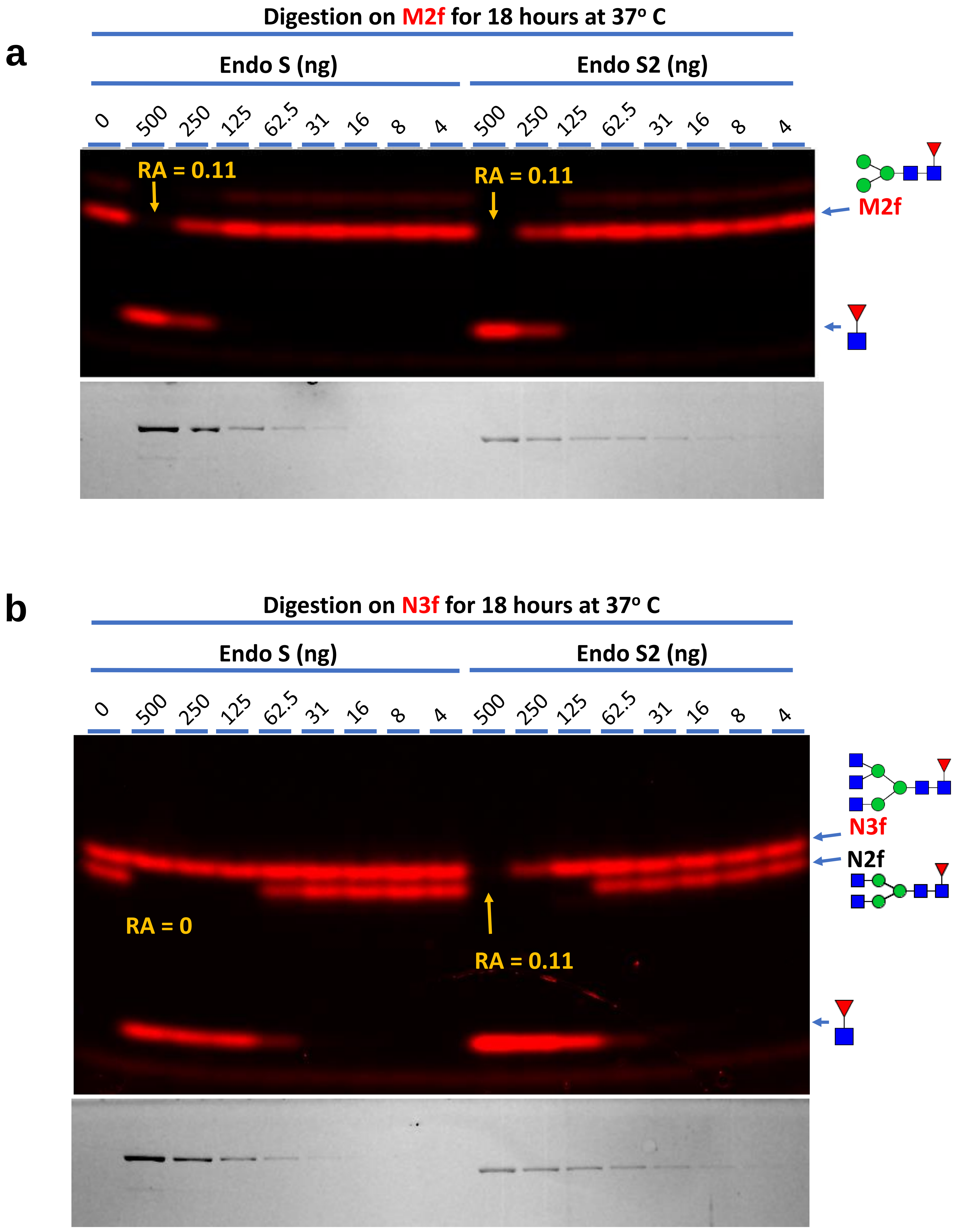

Supplemental Figure 4. Relative activity of Endo S/S2 on M2f and N3f.
All enzyme digestions were performed at 37° C for 18 hours and separated on 15% SDS-gels and imaged for glycans (upper panels) and protein (lower panels). **a** Assay on M2f. **b** Assay on N3f. Both enzymes showed complete digestion of M2f at the highest dosage in **a**. Prescence of N2f in **b** was due to incomplete conversion of N2f to N3f during synthesis. Endo S2 showed complete digestion of N3f at the highest dosage.

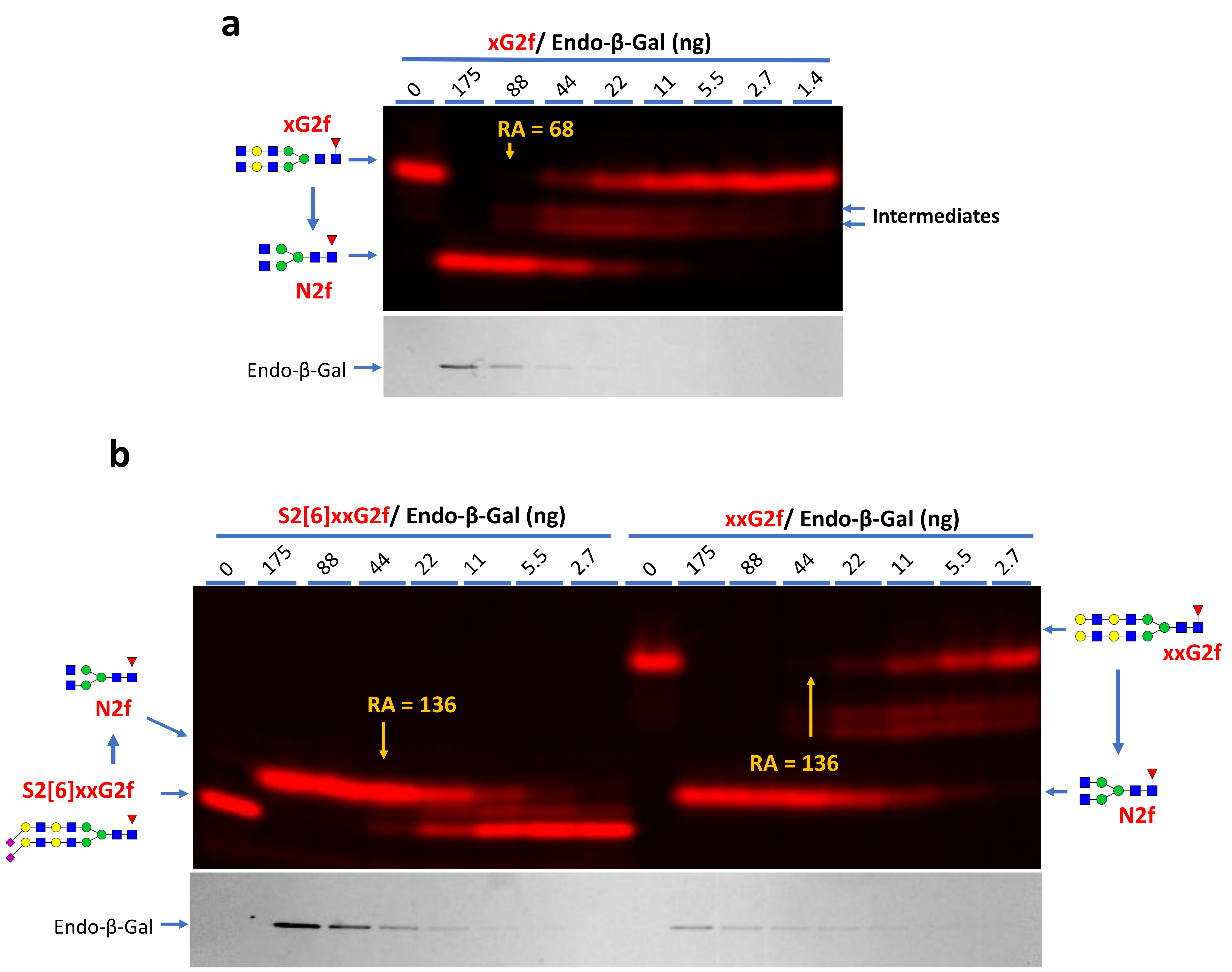

Supplemental Figure 5. The relative activity of Endo-β-Gal on different substrates.
In each reaction, 2 pmol of a substrate glycan was digested with indicated amount of Endo-β-Gal in 20 µl MES buffer pH 6.0 for 10 minutes at 37° C and then separated on 15% gel. Both glycan images and protein images are shown for the assays. **a** Assay on xG2f. **b** Assay on xxG2f and S2[6]xxG2f.
