# Supplementary material for: Endoglycosidase assay using enzymatically synthesized fluorophore-labeled glycans as substrates to uncover enzyme substrate specificities
Source: Commun Biol. 2022 May 25;5:501. doi: 10.1038/s42003-022-03444-3 (PMC9132957; doi:10.1038/s42003-022-03444-3)
Supplement: Supplementary file 3 — Description of Additional Supplementary Files [file 42003_2022_3444_MOESM3_ESM.pdf]

Description of Additional Supplementary Files

File name: Supplementary Data 1
Description: This zip file contains all unprocessed raw images of all the figures and supplemental figures.
