# Supplementary figures and images for: Endoglycosidase assay using enzymatically synthesized fluorophore-labeled glycans as substrates to uncover enzyme substrate specificities
Source: Commun Biol. 2022 May 25;5:501. doi: 10.1038/s42003-022-03444-3 (PMC9132957; doi:10.1038/s42003-022-03444-3)

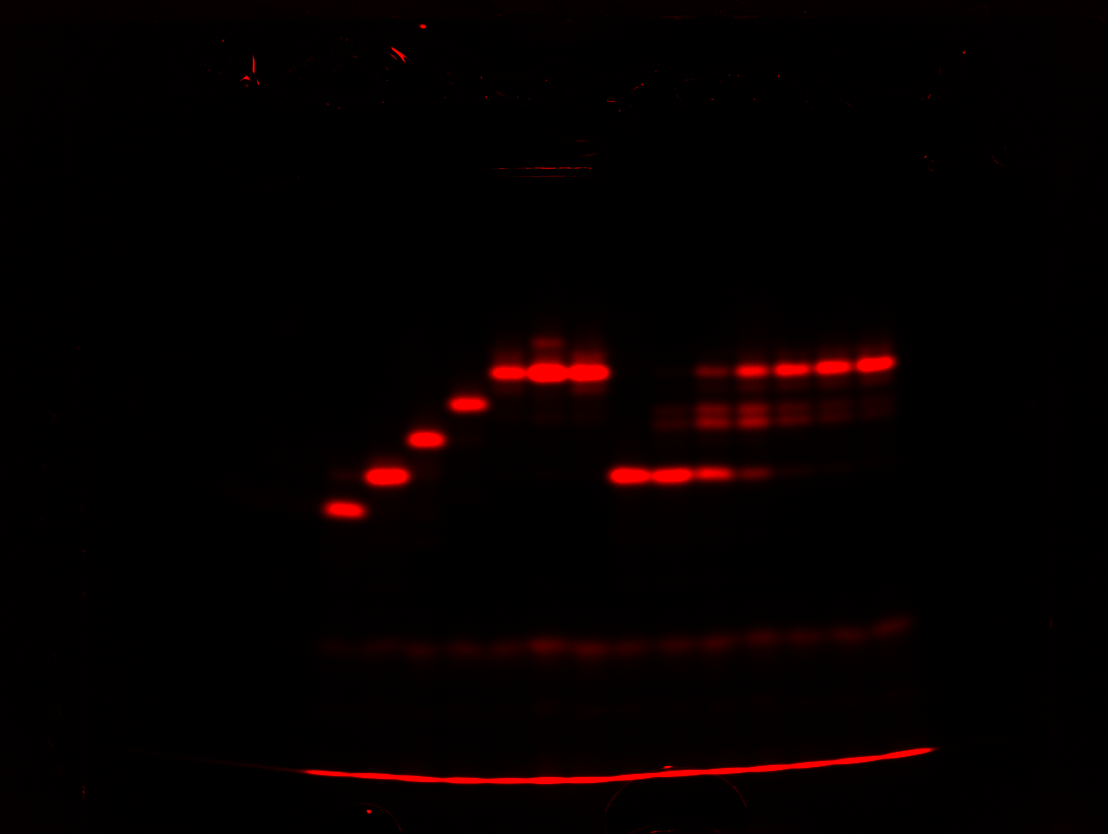

Supplement: Supplementary file 4 — Supplementary Data 1 [file 42003_2022_3444_MOESM4_ESM.zip › Unprocessed data/Fig. 1/Fig. 1-1.png]

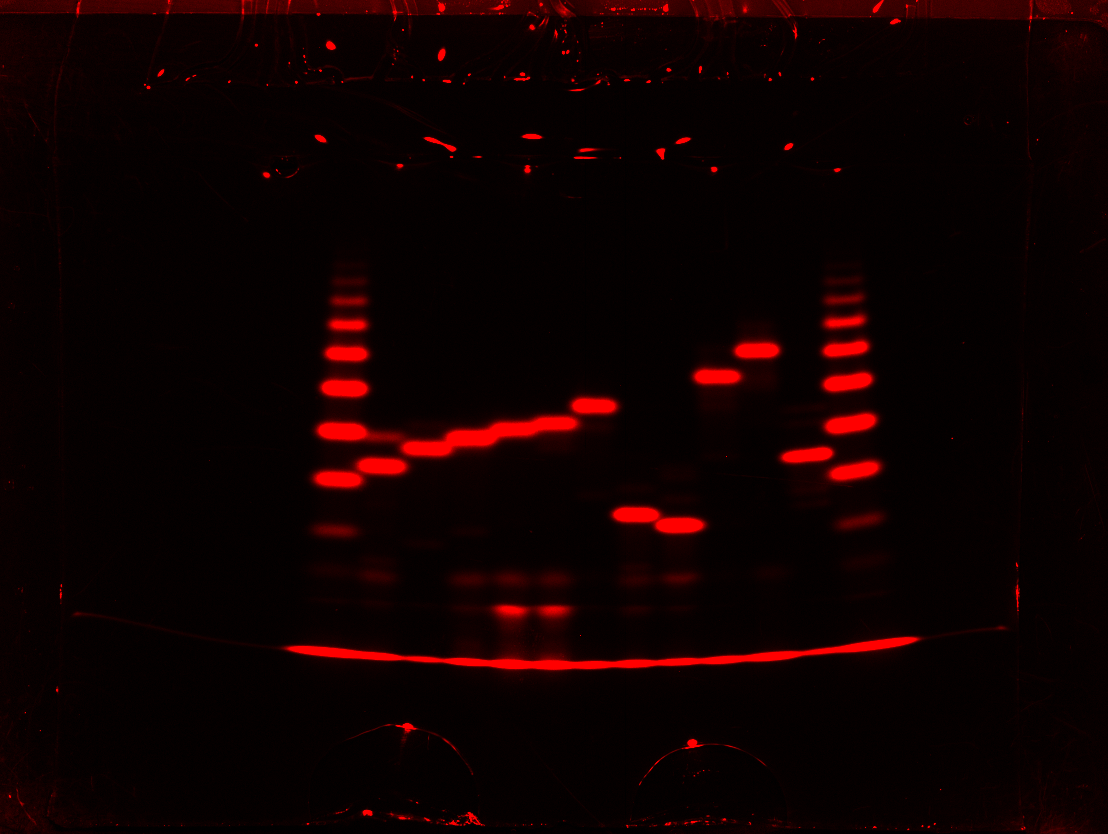

Supplement: Supplementary file 4 — Supplementary Data 1 [file 42003_2022_3444_MOESM4_ESM.zip › Unprocessed data/Fig. 2/Fig. 2.png]

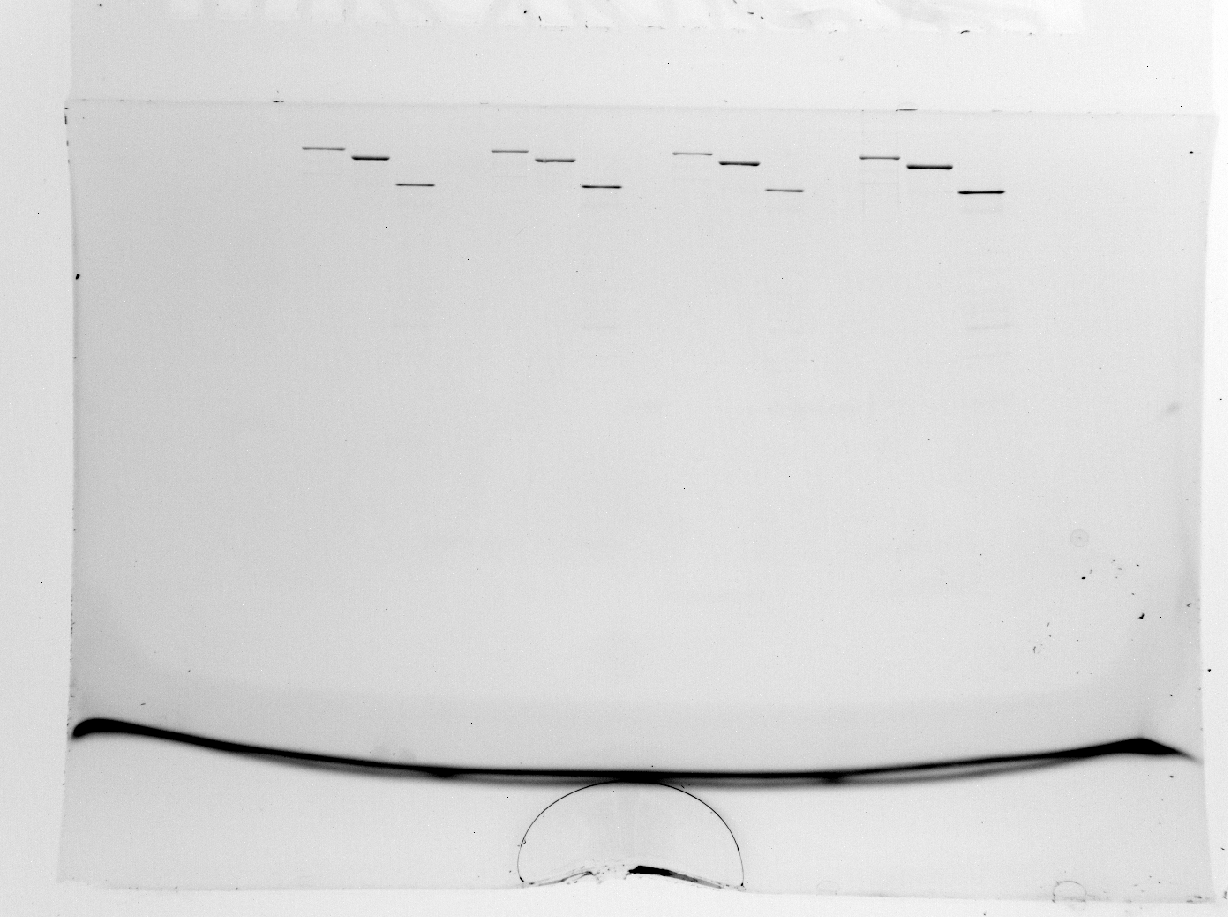

Supplement: Supplementary file 4 — Supplementary Data 1 [file 42003_2022_3444_MOESM4_ESM.zip › Unprocessed data/Fig. 3/Fig. 3B1-2.tif]

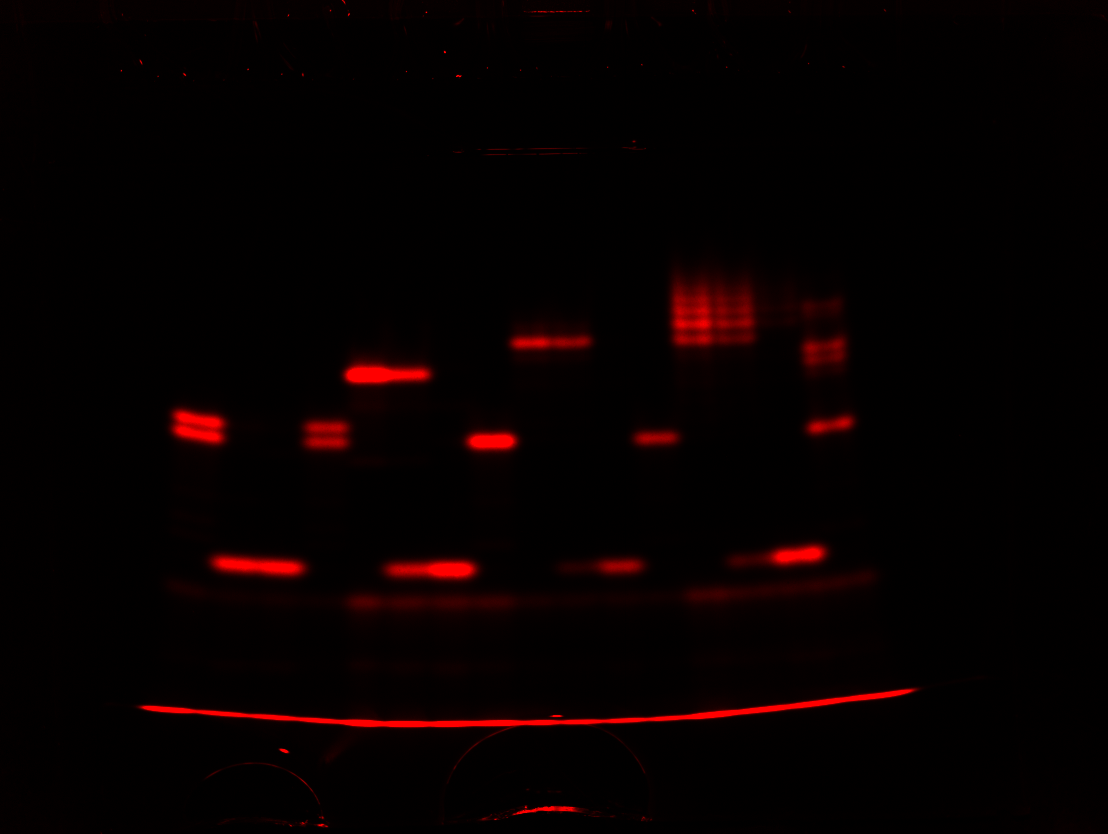

Supplement: Supplementary file 4 — Supplementary Data 1 [file 42003_2022_3444_MOESM4_ESM.zip › Unprocessed data/Fig. 3/Fig. 3B2.png]

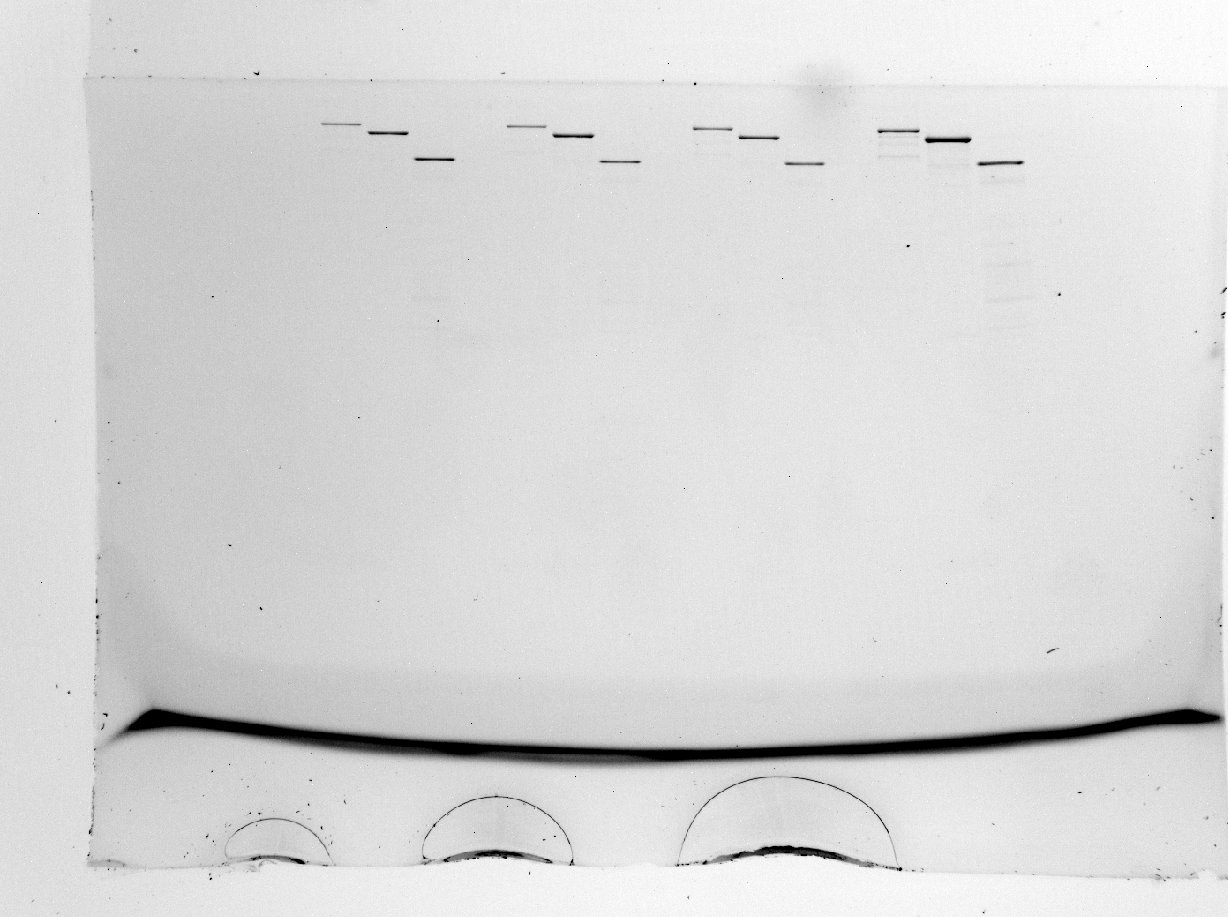

Supplement: Supplementary file 4 — Supplementary Data 1 [file 42003_2022_3444_MOESM4_ESM.zip › Unprocessed data/Fig. 3/Fig. 3B2-2.tif]

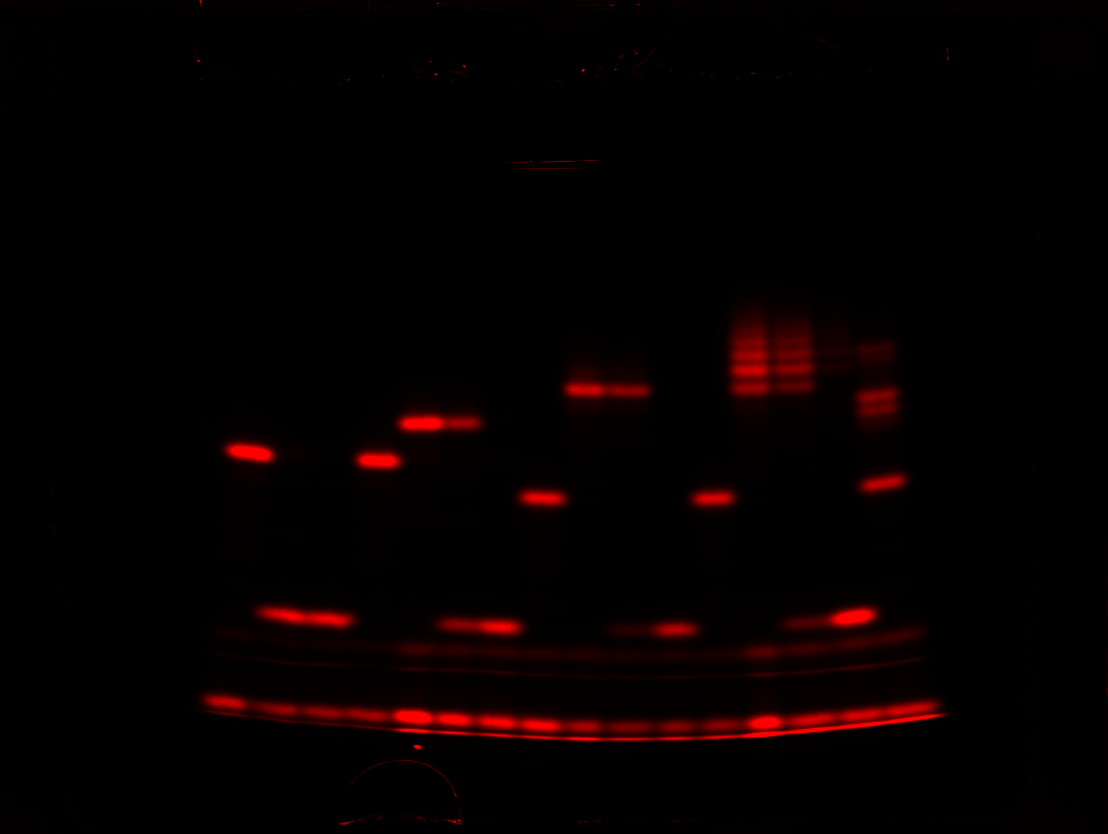

Supplement: Supplementary file 4 — Supplementary Data 1 [file 42003_2022_3444_MOESM4_ESM.zip › Unprocessed data/Fig. 3/Fig. 3B3.png]

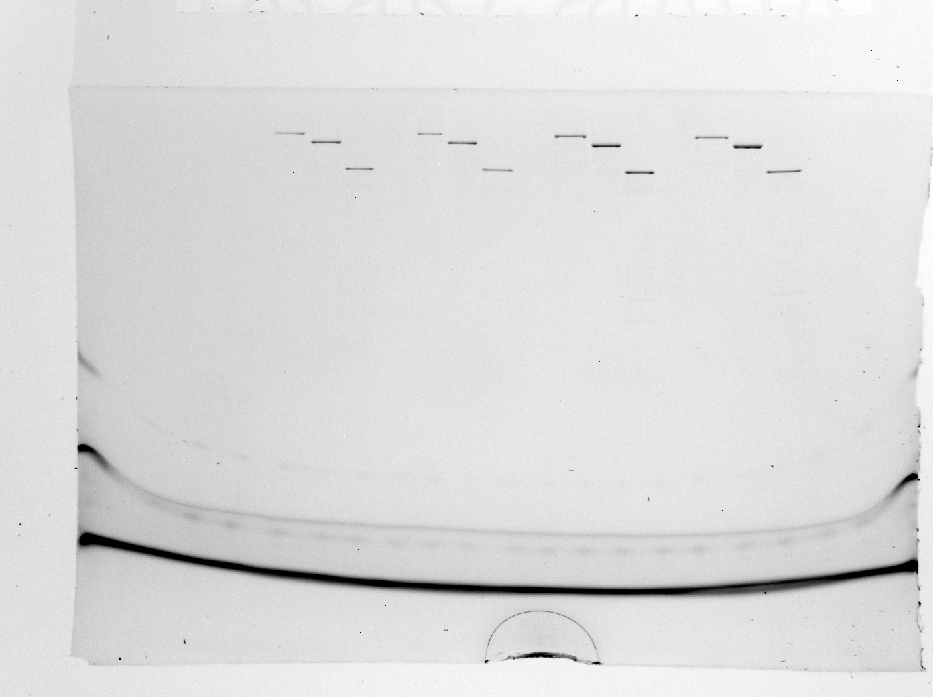

Supplement: Supplementary file 4 — Supplementary Data 1 [file 42003_2022_3444_MOESM4_ESM.zip › Unprocessed data/Fig. 3/Fig. 3B3-2.tif]

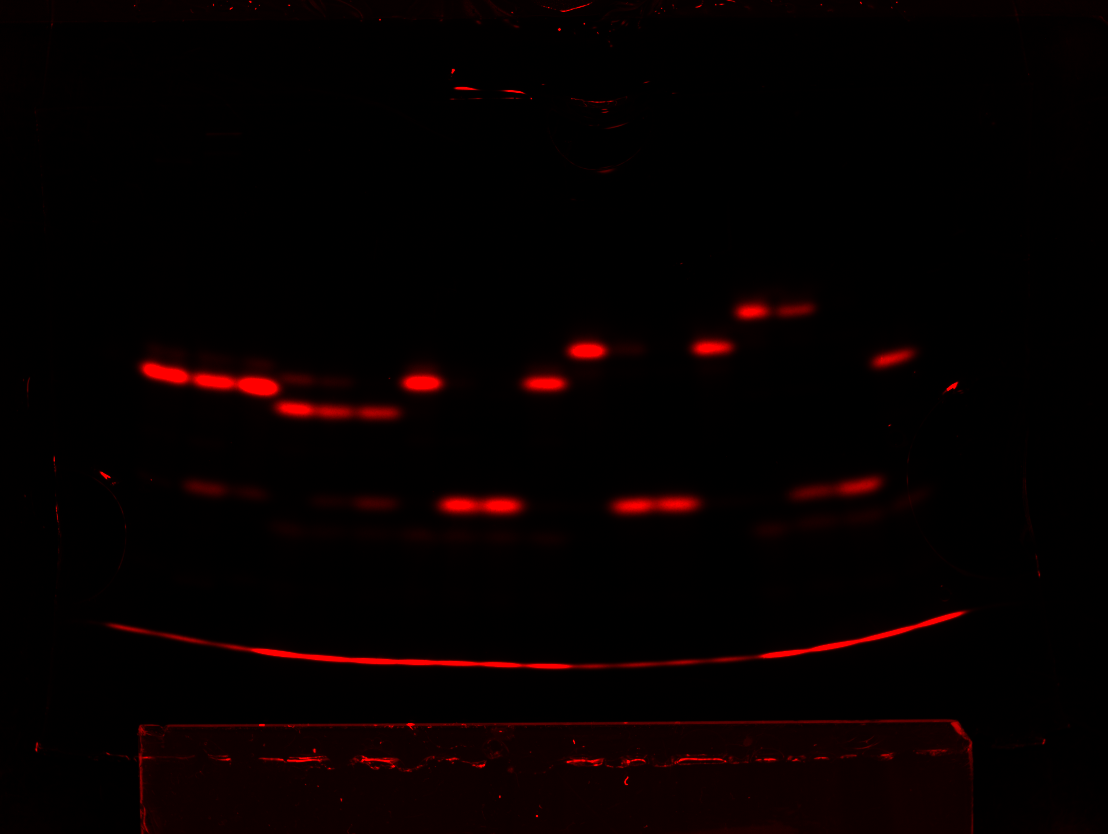

Supplement: Supplementary file 4 — Supplementary Data 1 [file 42003_2022_3444_MOESM4_ESM.zip › Unprocessed data/Fig. 3/Fig. 3C.png]

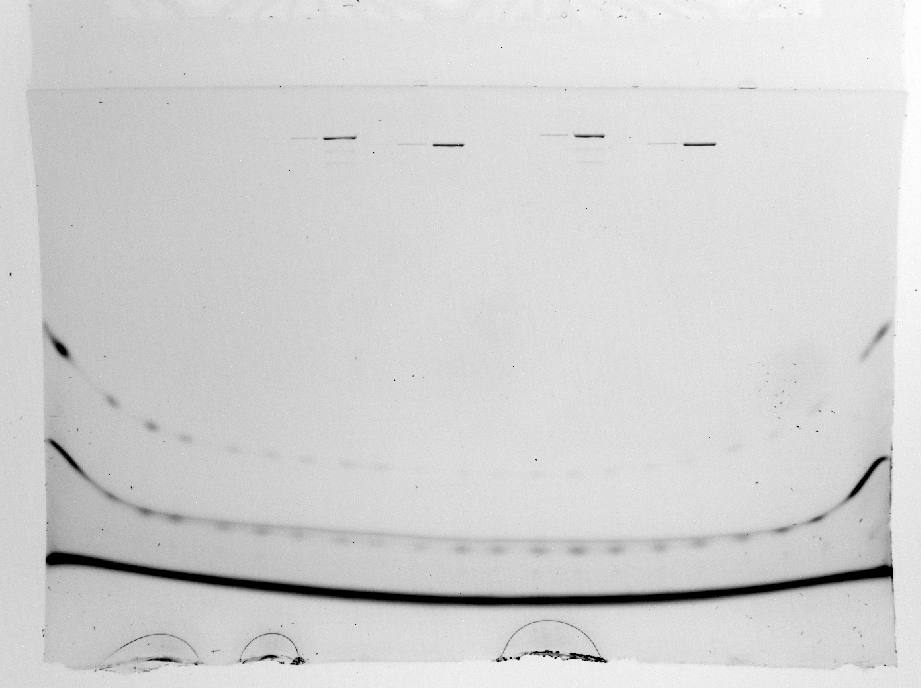

Supplement: Supplementary file 4 — Supplementary Data 1 [file 42003_2022_3444_MOESM4_ESM.zip › Unprocessed data/Fig. 3/Fig. 3C2.tif]

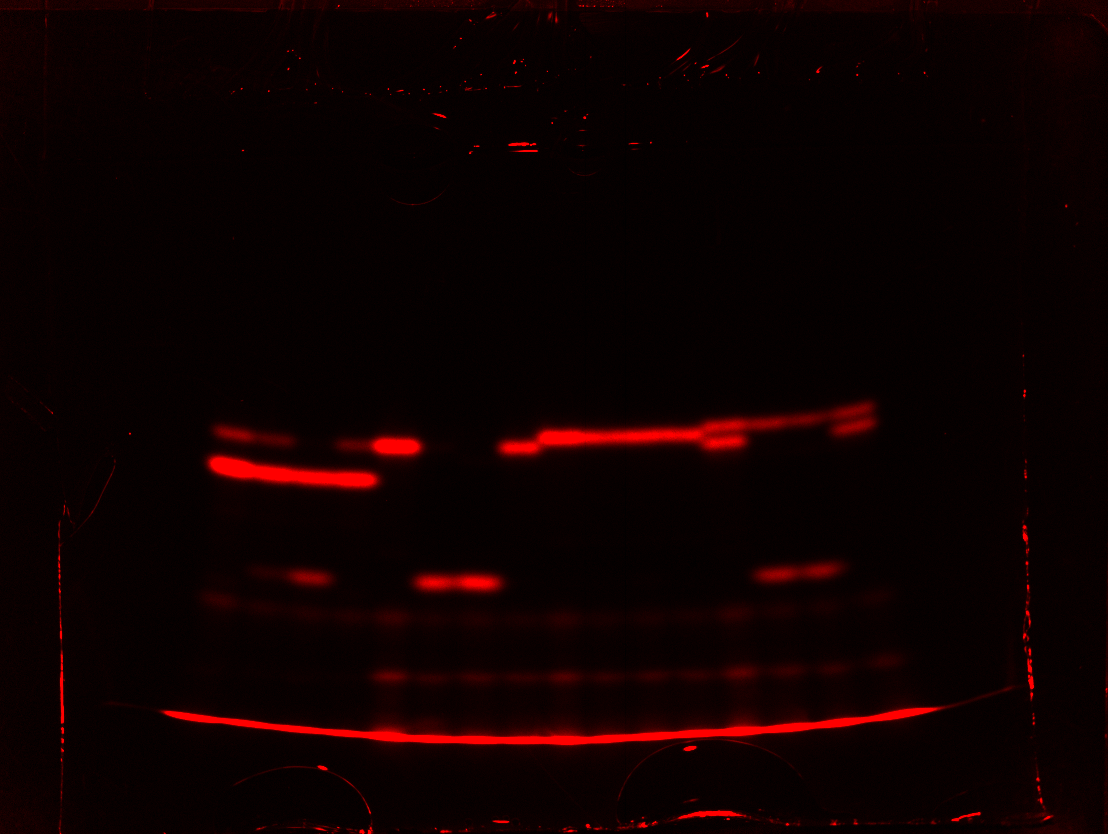

Supplement: Supplementary file 4 — Supplementary Data 1 [file 42003_2022_3444_MOESM4_ESM.zip › Unprocessed data/Fig. 3/Fig. 3D.png]

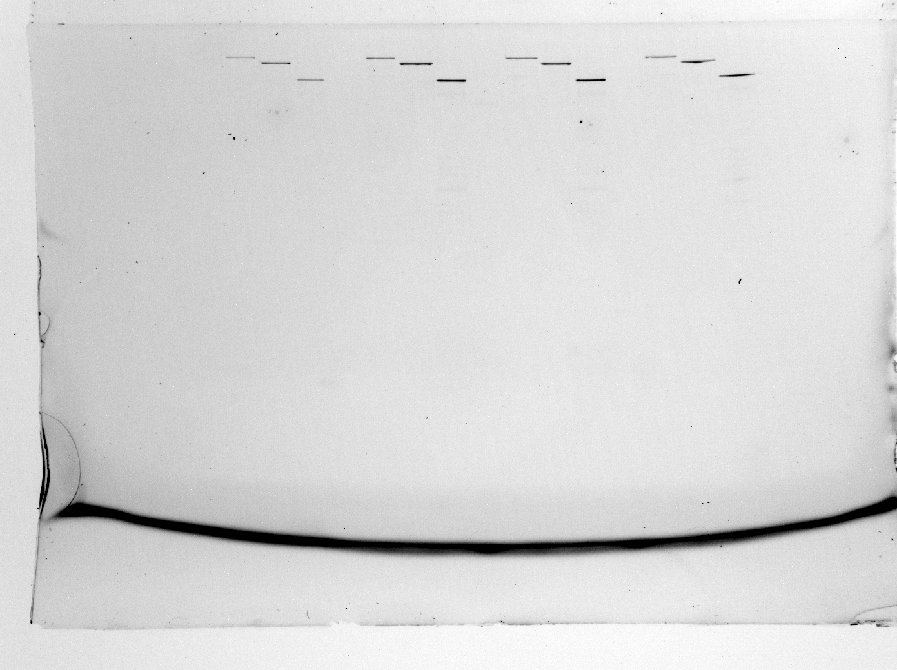

Supplement: Supplementary file 4 — Supplementary Data 1 [file 42003_2022_3444_MOESM4_ESM.zip › Unprocessed data/Fig. 3/Fig. 3D-2.tif]

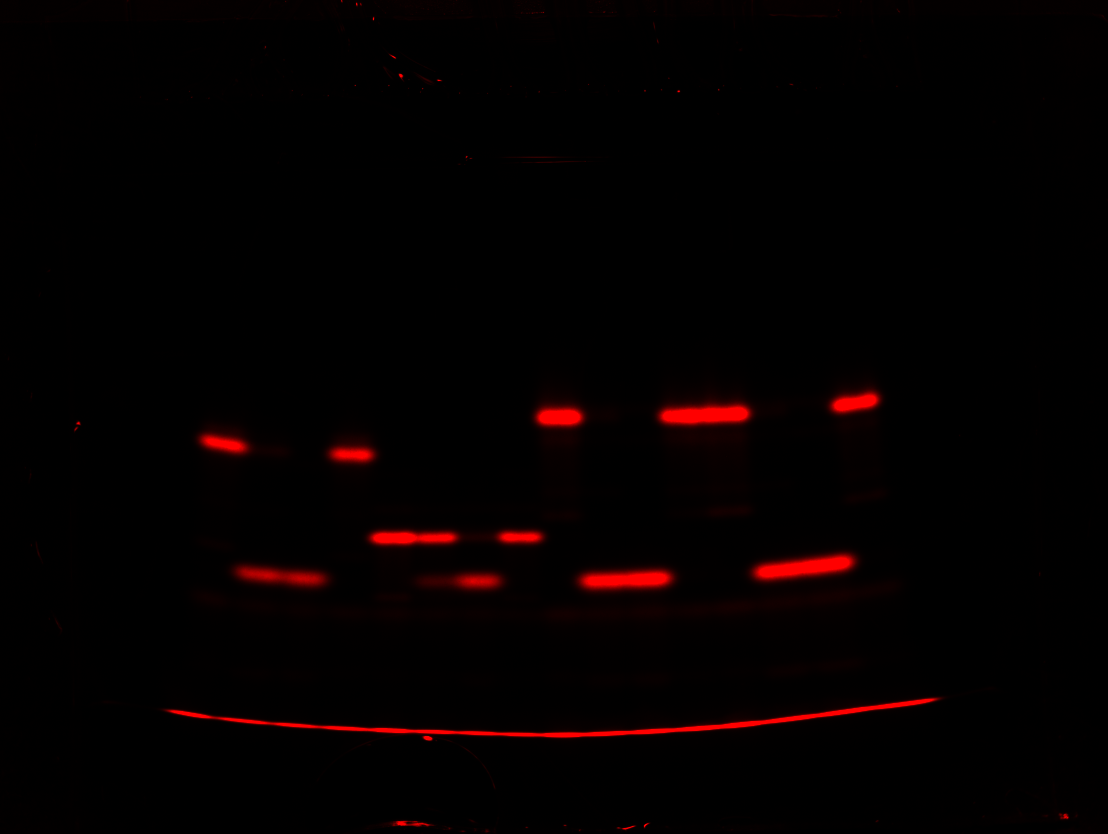

Supplement: Supplementary file 4 — Supplementary Data 1 [file 42003_2022_3444_MOESM4_ESM.zip › Unprocessed data/Fig. 3/Fig.3B1.png]

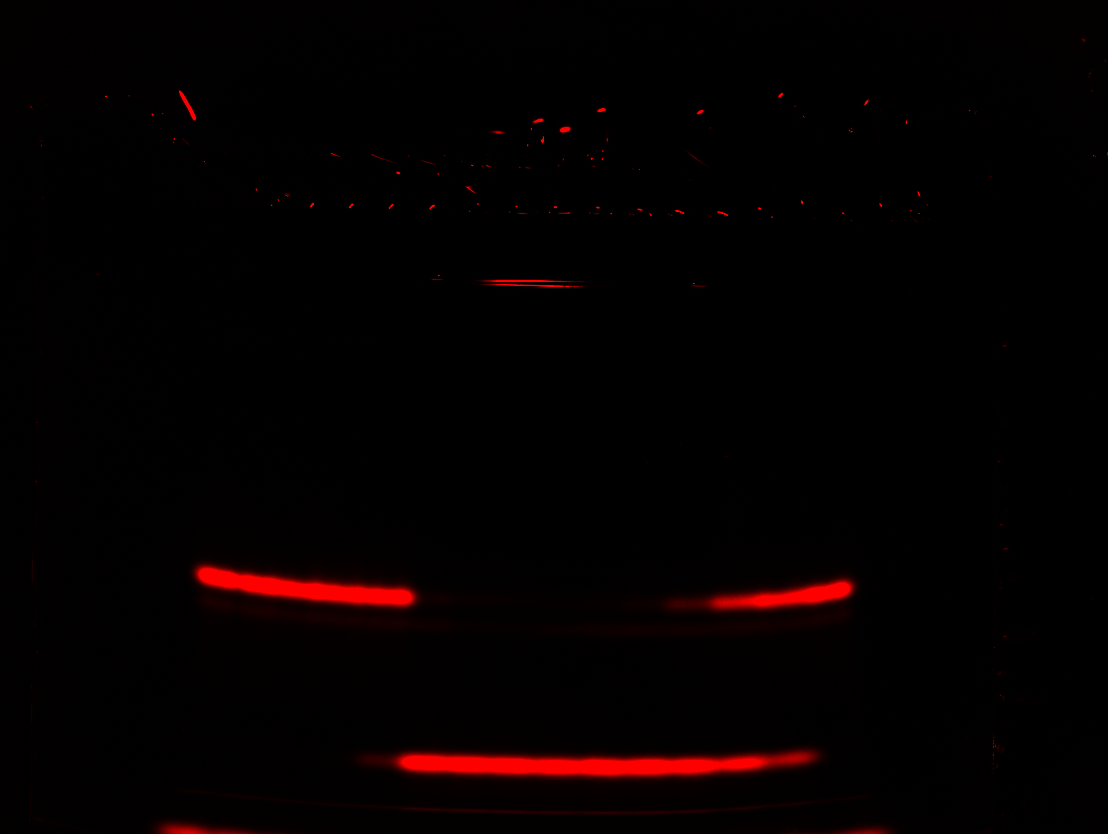

Supplement: Supplementary file 4 — Supplementary Data 1 [file 42003_2022_3444_MOESM4_ESM.zip › Unprocessed data/Fig. 4/S2A1.png]

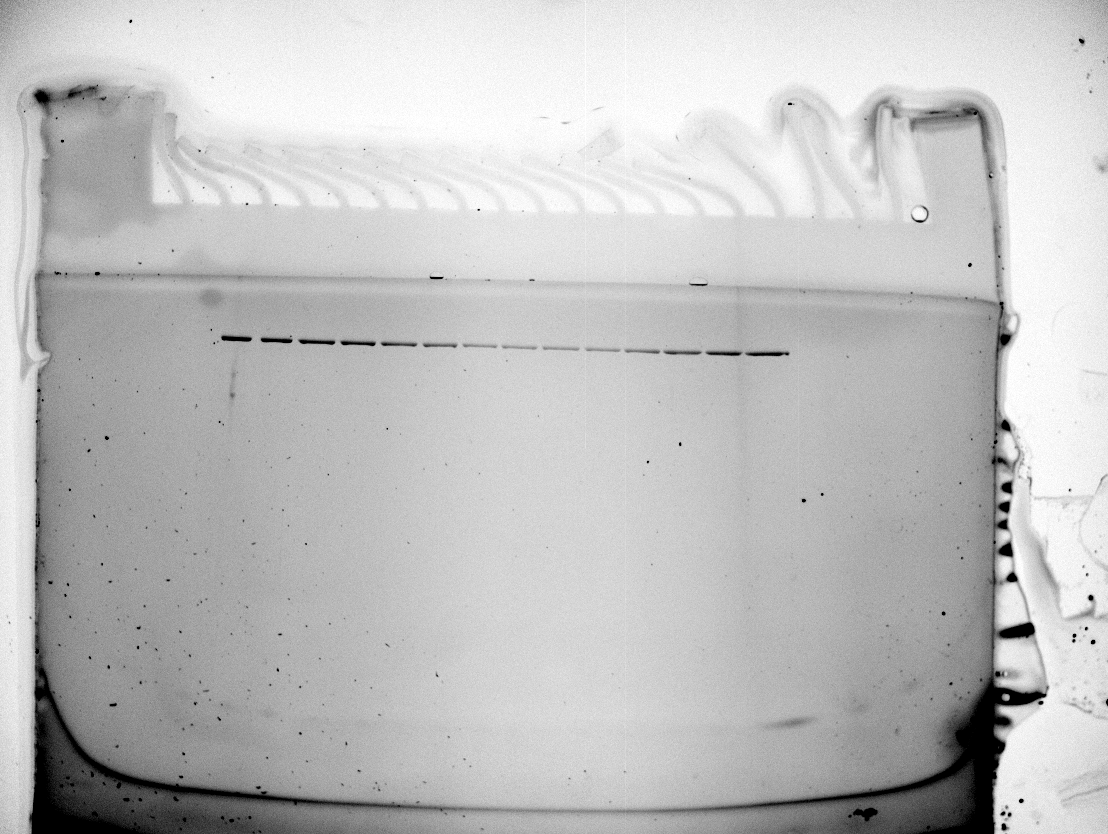

Supplement: Supplementary file 4 — Supplementary Data 1 [file 42003_2022_3444_MOESM4_ESM.zip › Unprocessed data/Fig. 4/S2A2.png]

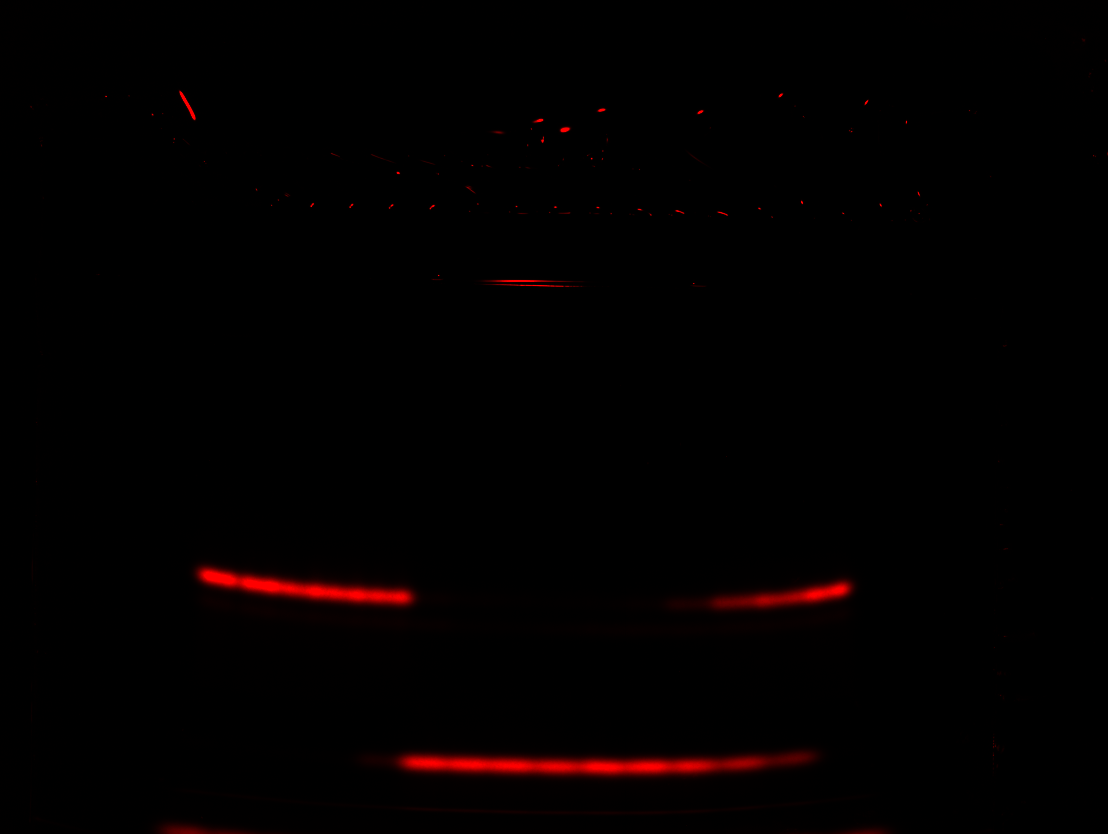

Supplement: Supplementary file 4 — Supplementary Data 1 [file 42003_2022_3444_MOESM4_ESM.zip › Unprocessed data/Fig. 4/S2B1.png]

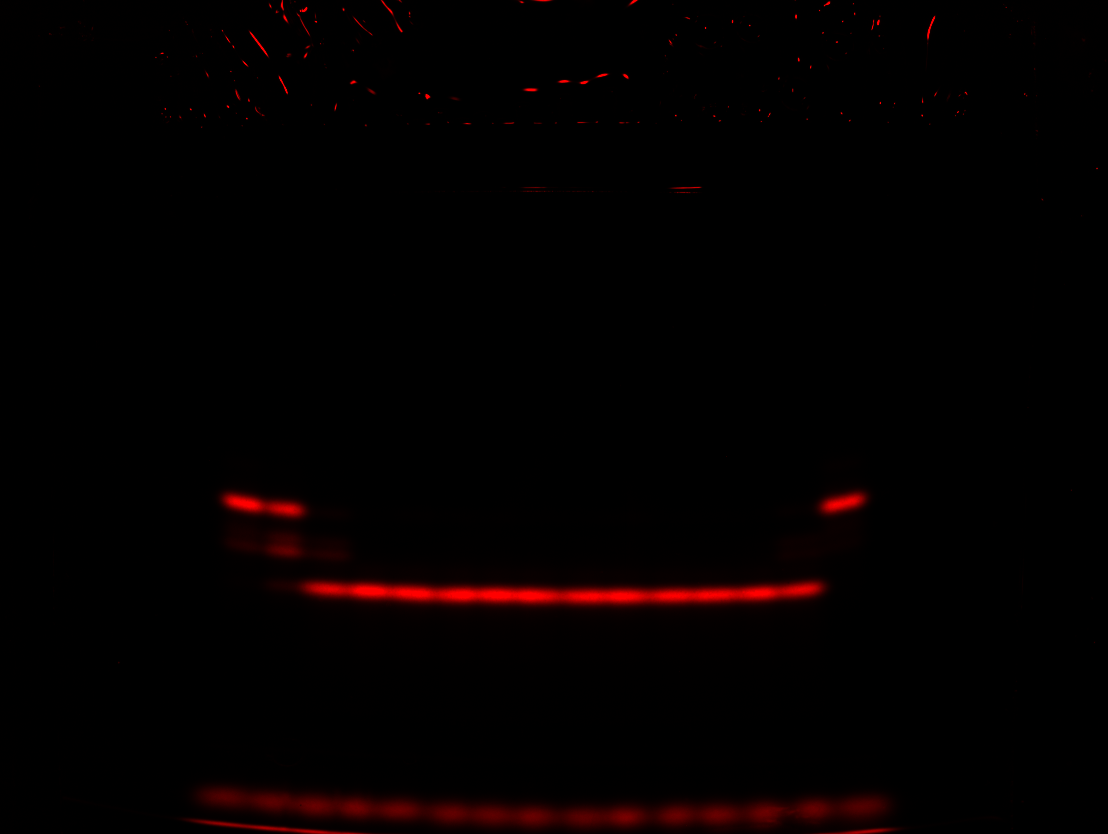

Supplement: Supplementary file 4 — Supplementary Data 1 [file 42003_2022_3444_MOESM4_ESM.zip › Unprocessed data/Fig. 4/S2C1.png]

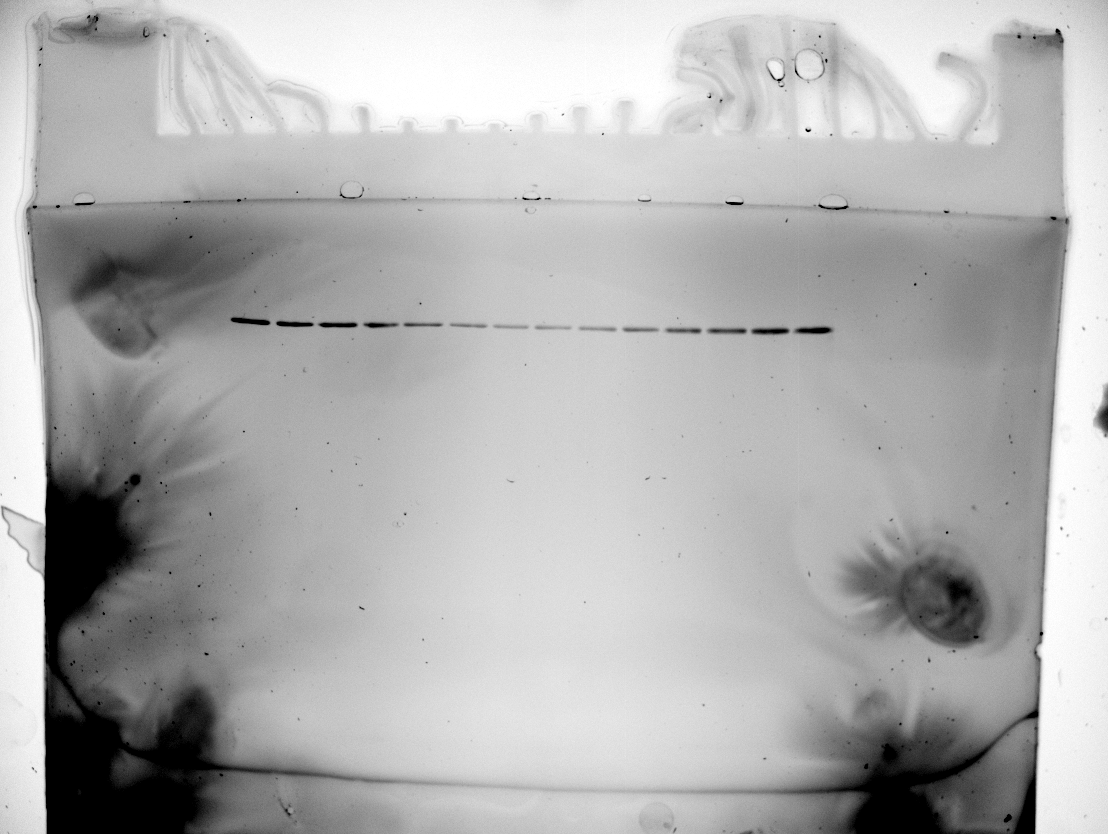

Supplement: Supplementary file 4 — Supplementary Data 1 [file 42003_2022_3444_MOESM4_ESM.zip › Unprocessed data/Fig. 4/S2C2.png]

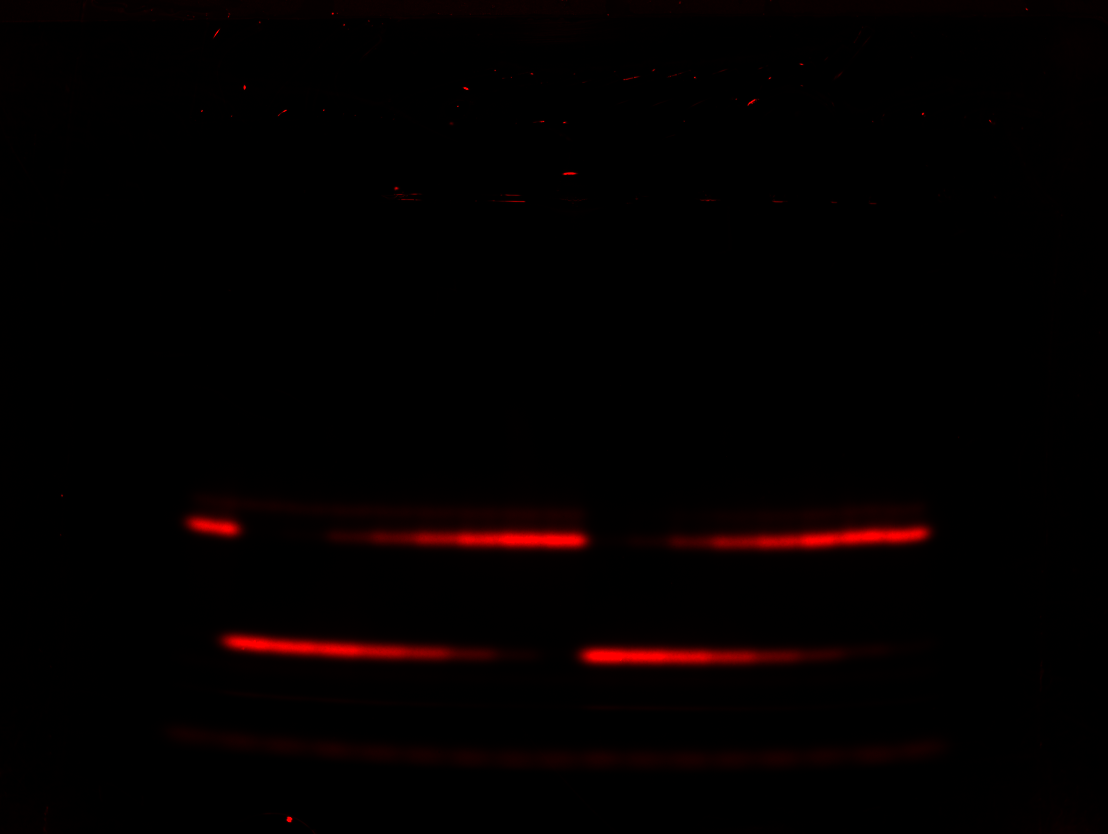

Supplement: Supplementary file 4 — Supplementary Data 1 [file 42003_2022_3444_MOESM4_ESM.zip › Unprocessed data/Fig. 5/F4A.png]

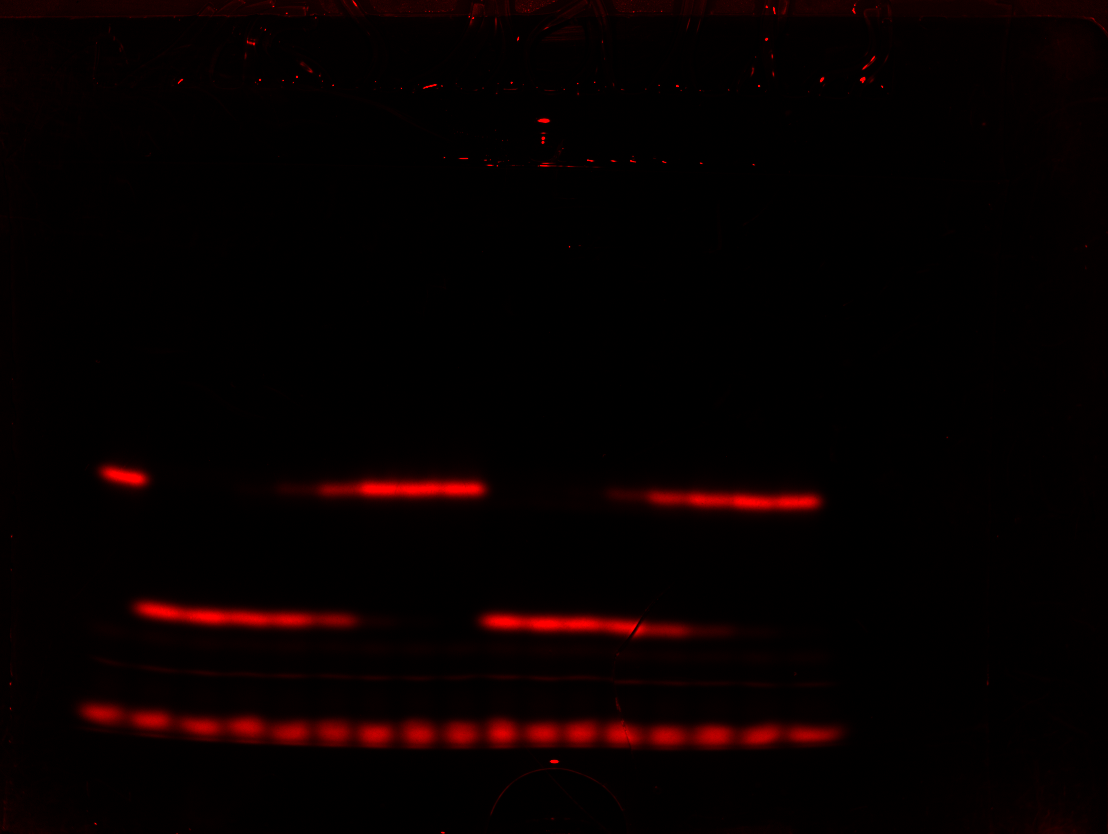

Supplement: Supplementary file 4 — Supplementary Data 1 [file 42003_2022_3444_MOESM4_ESM.zip › Unprocessed data/Fig. 5/F4B.png]

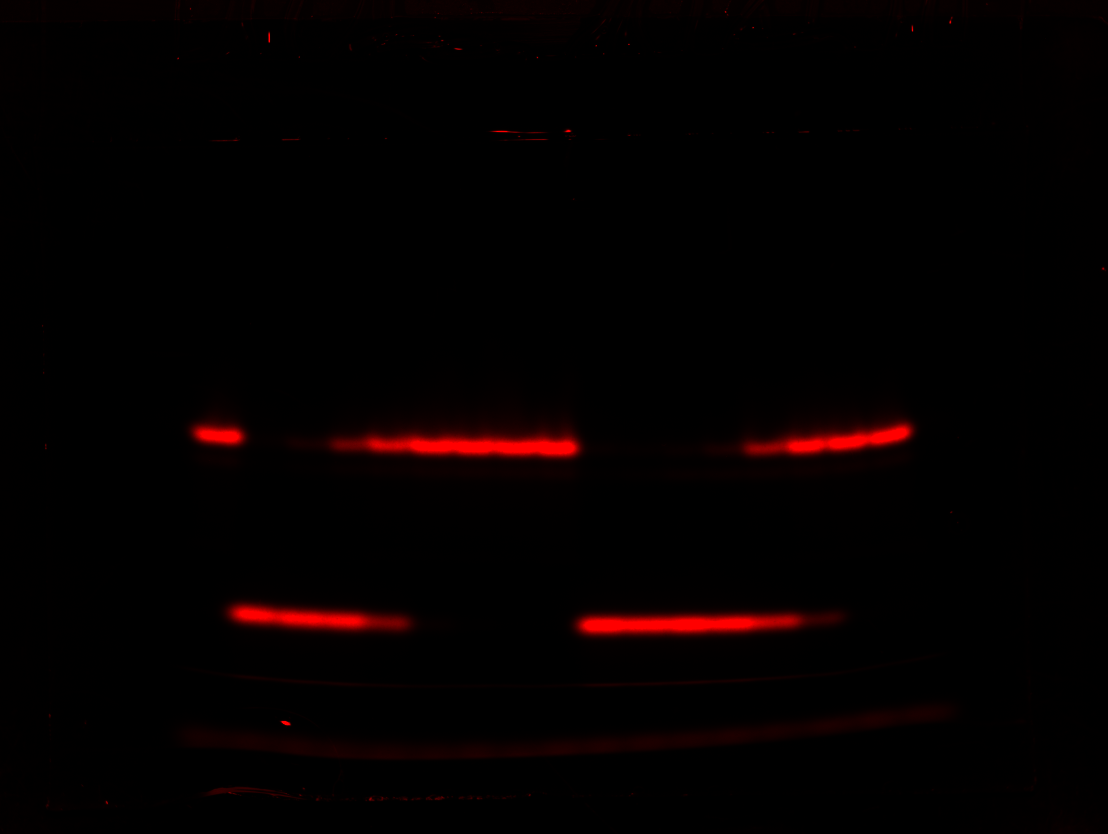

Supplement: Supplementary file 4 — Supplementary Data 1 [file 42003_2022_3444_MOESM4_ESM.zip › Unprocessed data/Fig. 5/F4C.png]

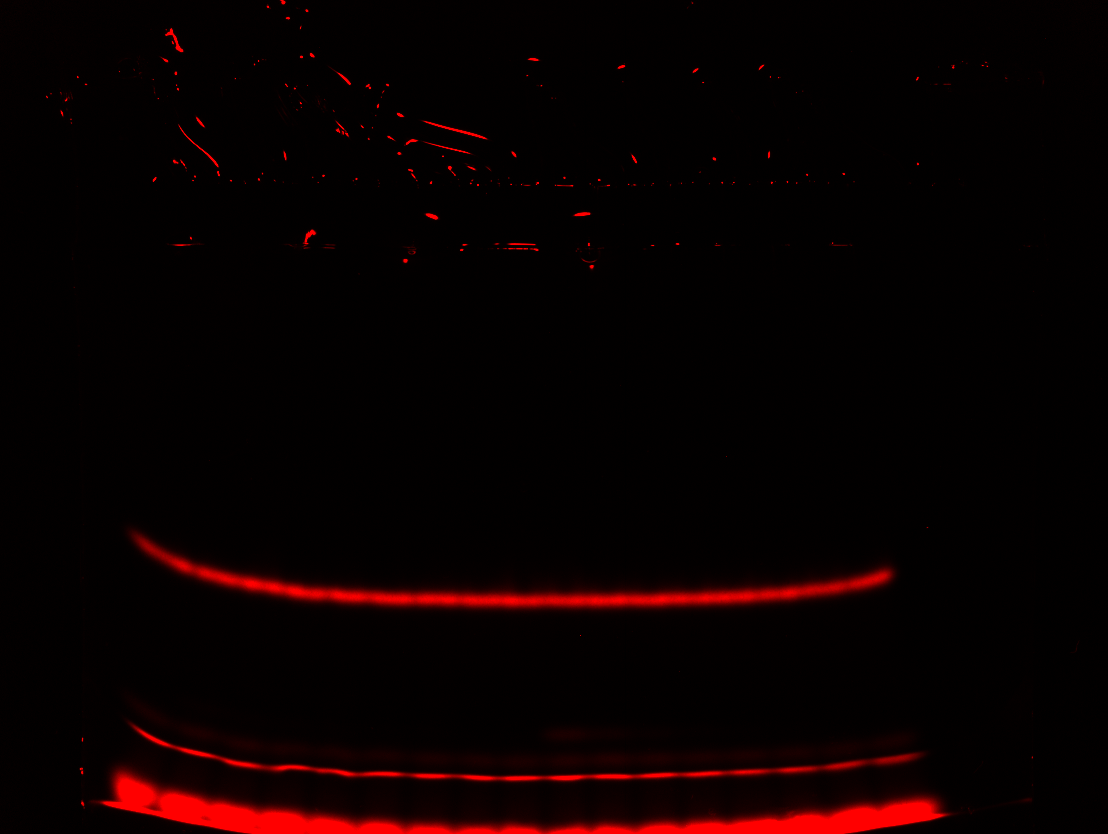

Supplement: Supplementary file 4 — Supplementary Data 1 [file 42003_2022_3444_MOESM4_ESM.zip › Unprocessed data/Fig. 5/F4D.png]

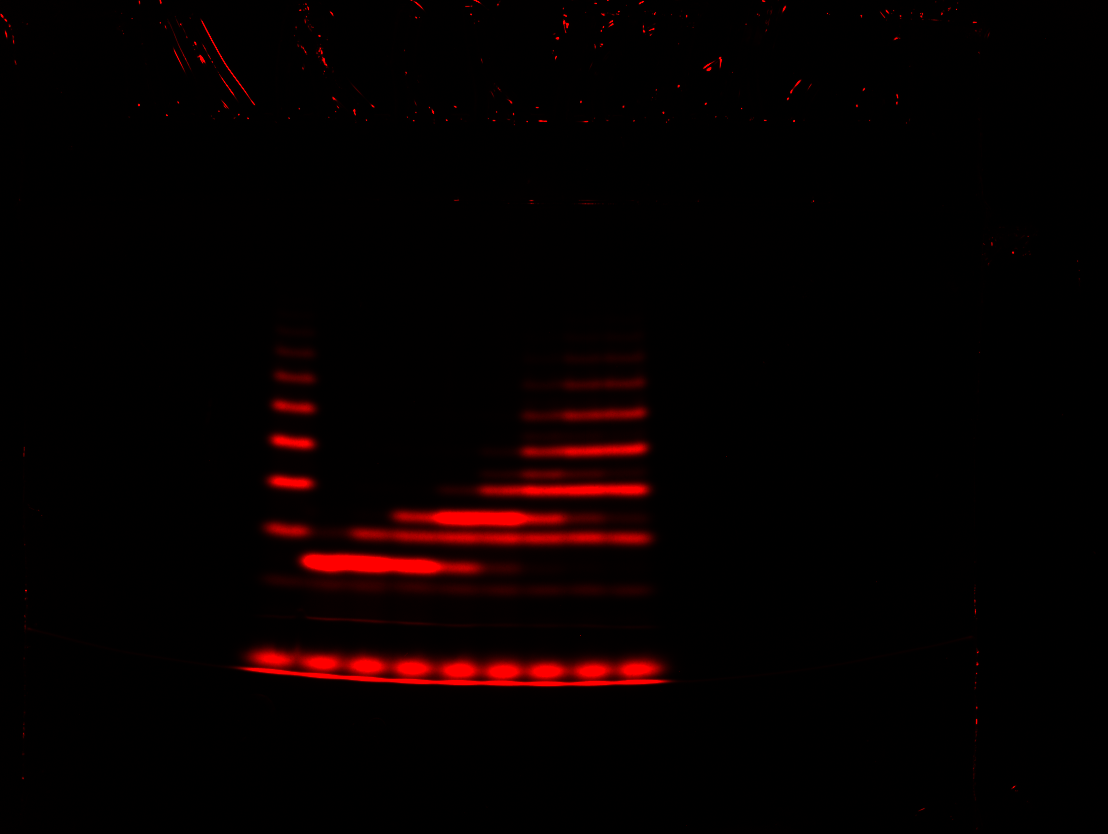

Supplement: Supplementary file 4 — Supplementary Data 1 [file 42003_2022_3444_MOESM4_ESM.zip › Unprocessed data/Fig. 7/Fig. 6.png]

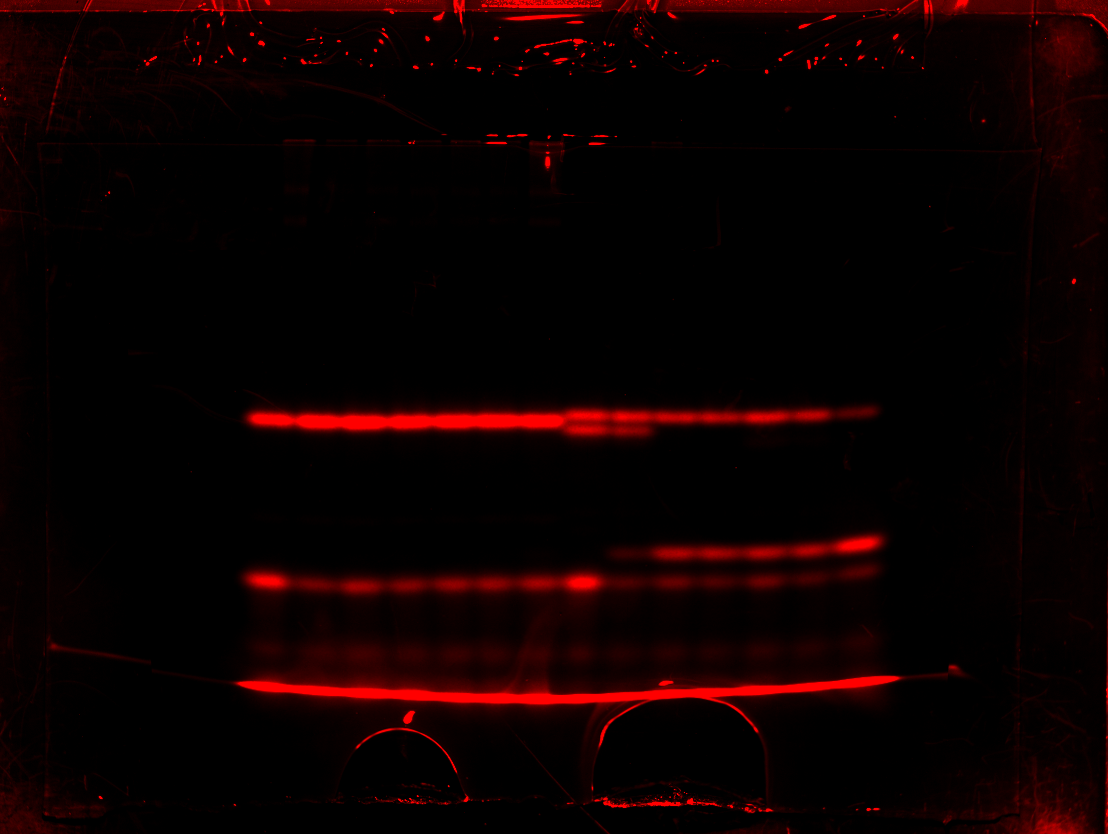

Supplement: Supplementary file 4 — Supplementary Data 1 [file 42003_2022_3444_MOESM4_ESM.zip › Unprocessed data/S Fig. 1/s Fig. 1C.png]

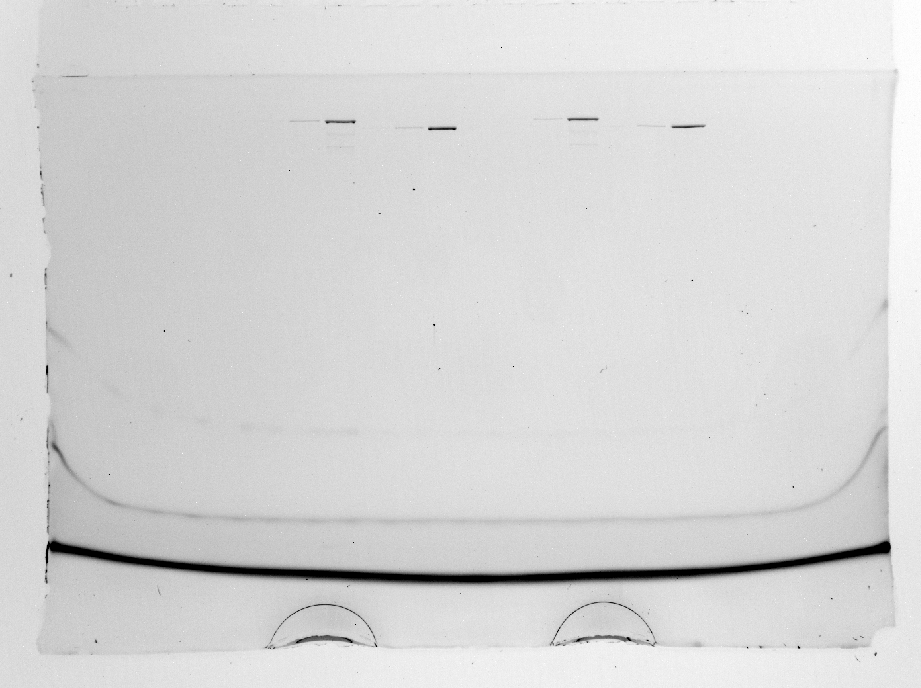

Supplement: Supplementary file 4 — Supplementary Data 1 [file 42003_2022_3444_MOESM4_ESM.zip › Unprocessed data/S Fig. 1/s Fig. 1A protein image.tif]

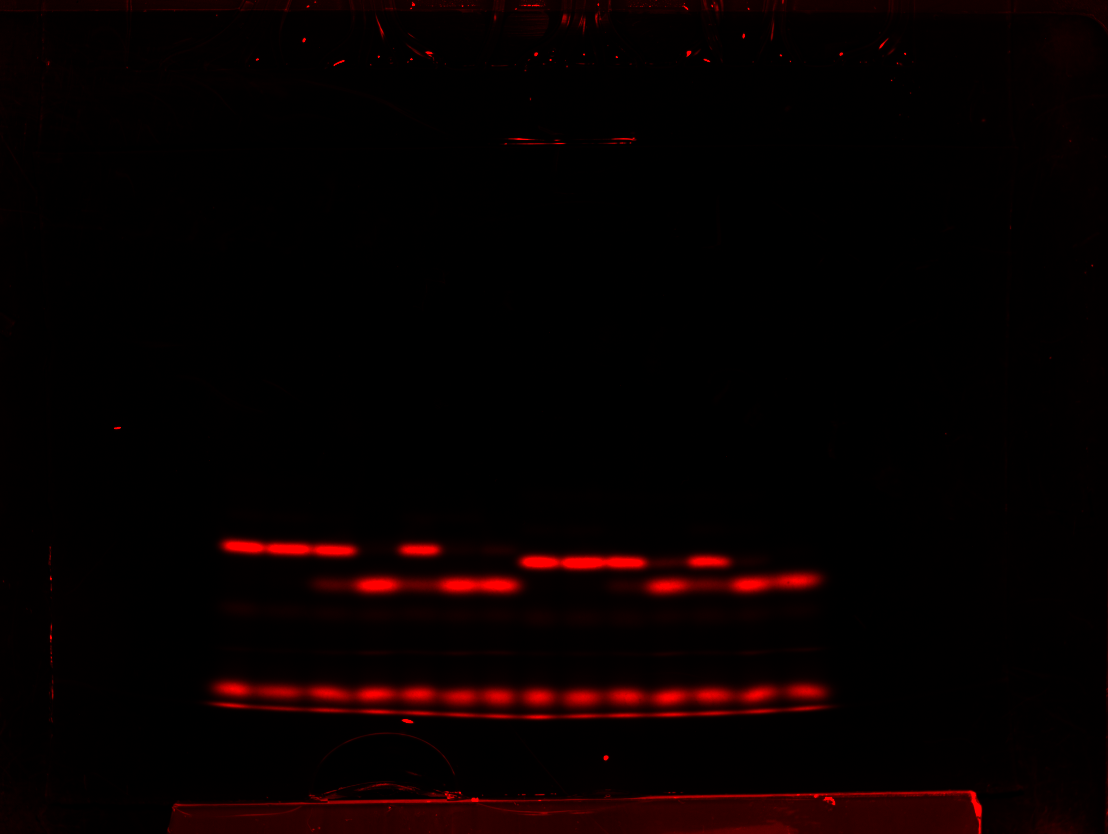

Supplement: Supplementary file 4 — Supplementary Data 1 [file 42003_2022_3444_MOESM4_ESM.zip › Unprocessed data/S Fig. 1/S Fig. 1A.png]

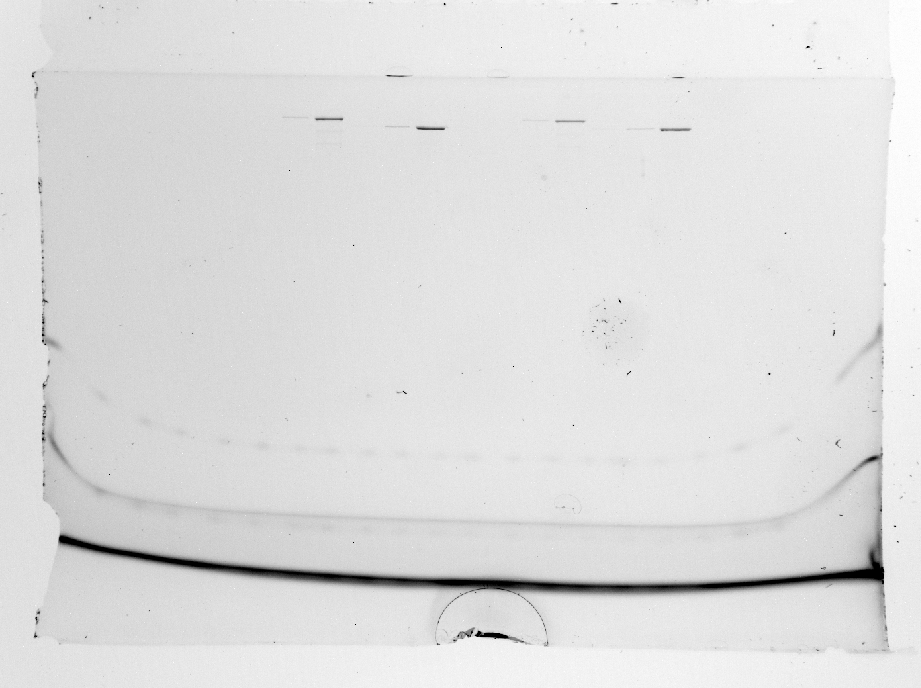

Supplement: Supplementary file 4 — Supplementary Data 1 [file 42003_2022_3444_MOESM4_ESM.zip › Unprocessed data/S Fig. 1/S Fig. 1B protein image.tif]

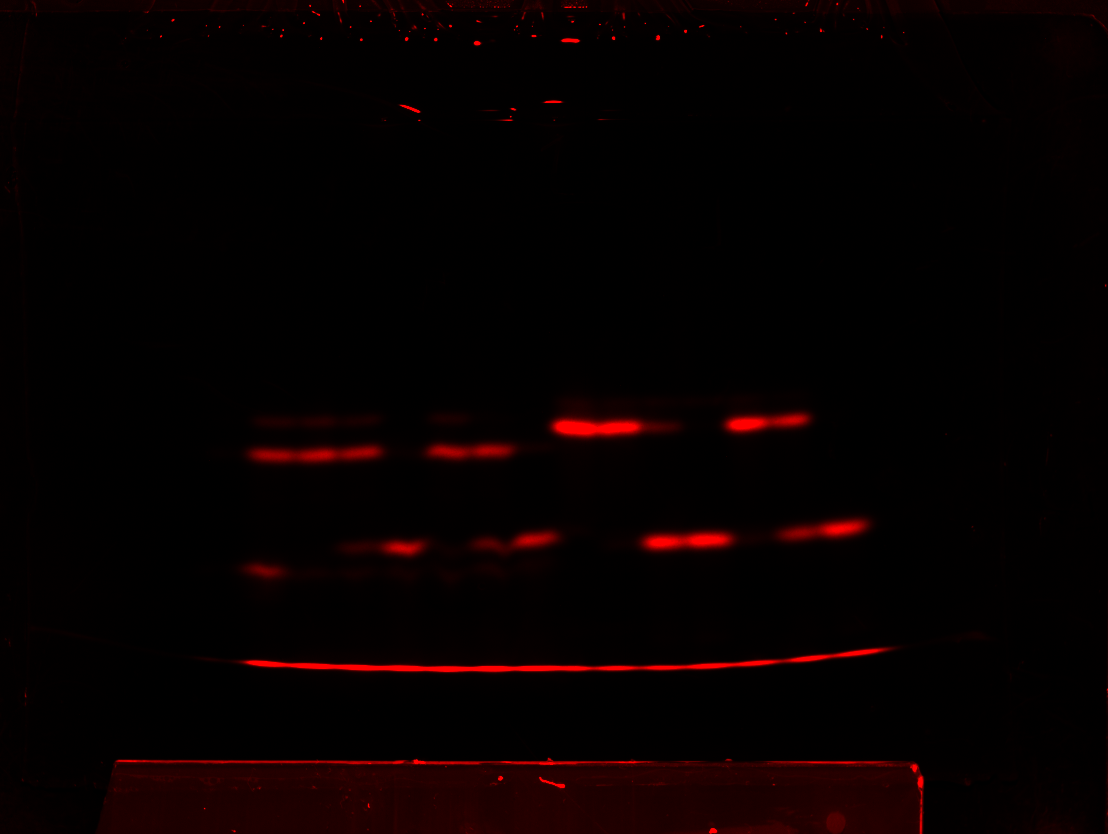

Supplement: Supplementary file 4 — Supplementary Data 1 [file 42003_2022_3444_MOESM4_ESM.zip › Unprocessed data/S Fig. 1/S Fig. 1B.png]

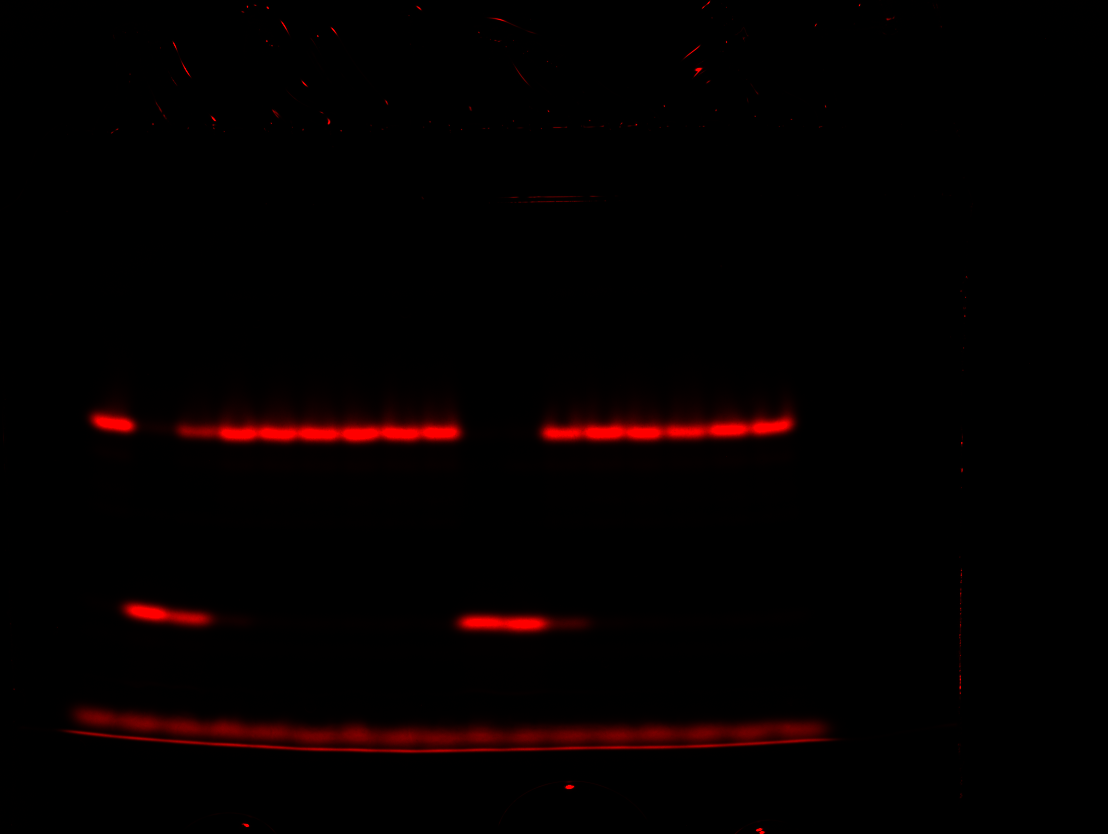

Supplement: Supplementary file 4 — Supplementary Data 1 [file 42003_2022_3444_MOESM4_ESM.zip › Unprocessed data/S Fig. 2/S Fig3A.png]

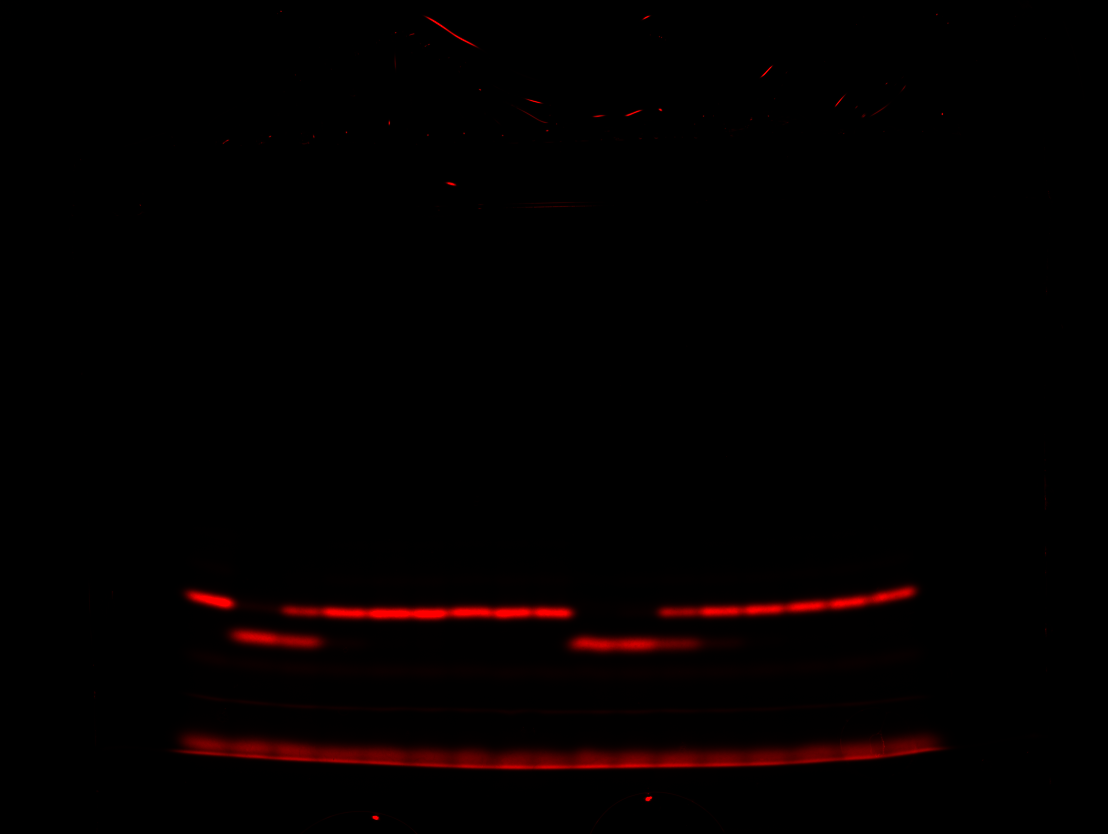

Supplement: Supplementary file 4 — Supplementary Data 1 [file 42003_2022_3444_MOESM4_ESM.zip › Unprocessed data/S Fig. 2/S Fig3B.png]

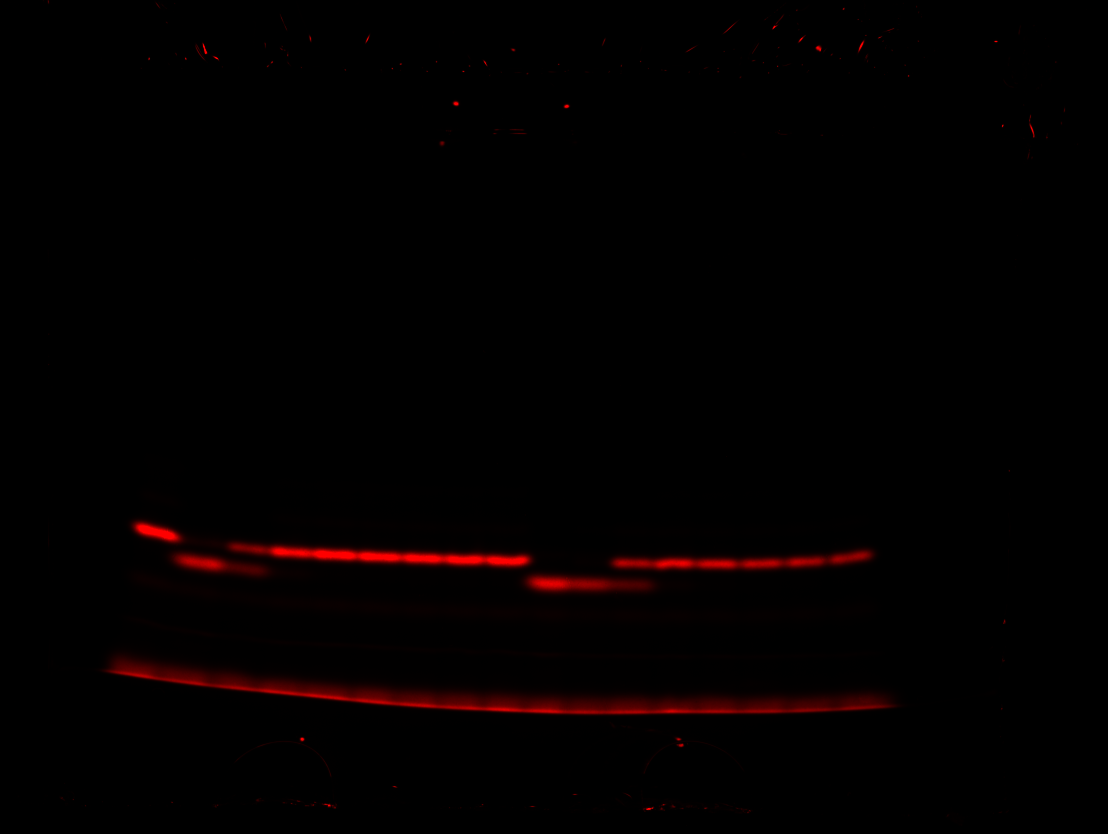

Supplement: Supplementary file 4 — Supplementary Data 1 [file 42003_2022_3444_MOESM4_ESM.zip › Unprocessed data/S Fig. 2/S Fig3C.png]

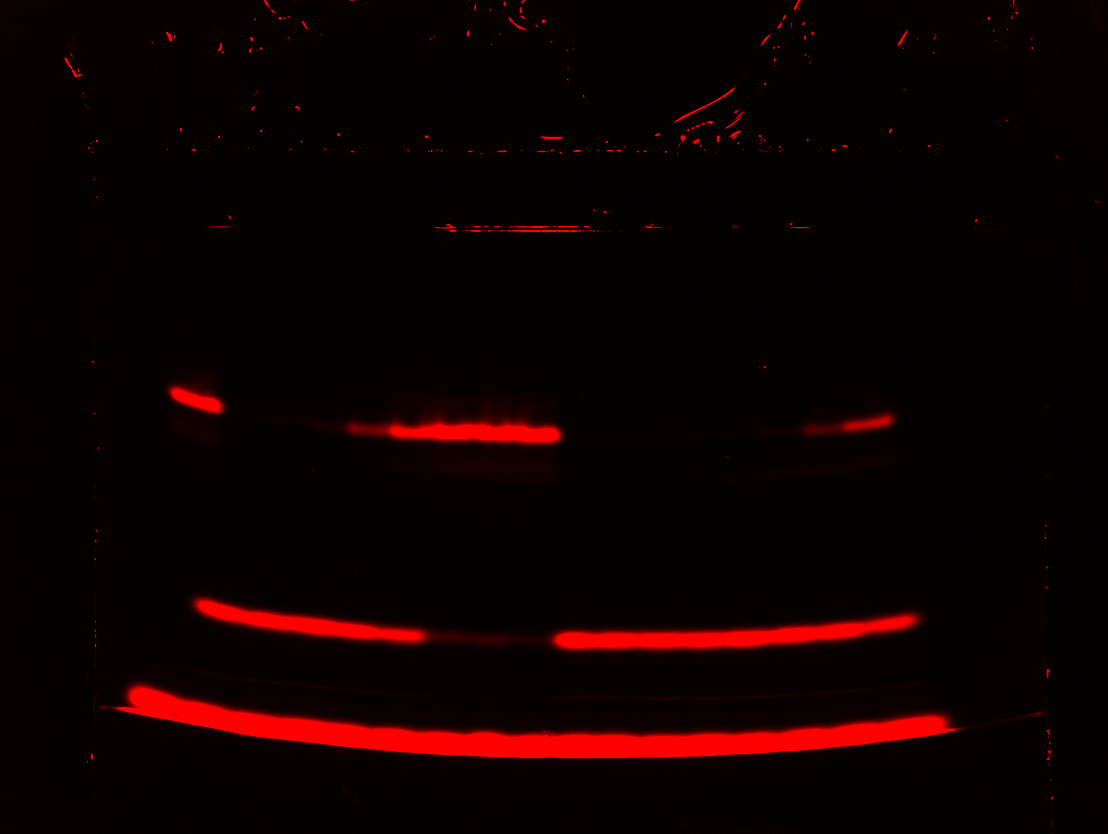

Supplement: Supplementary file 4 — Supplementary Data 1 [file 42003_2022_3444_MOESM4_ESM.zip › Unprocessed data/S Fig. 3/S Fig4A.png]

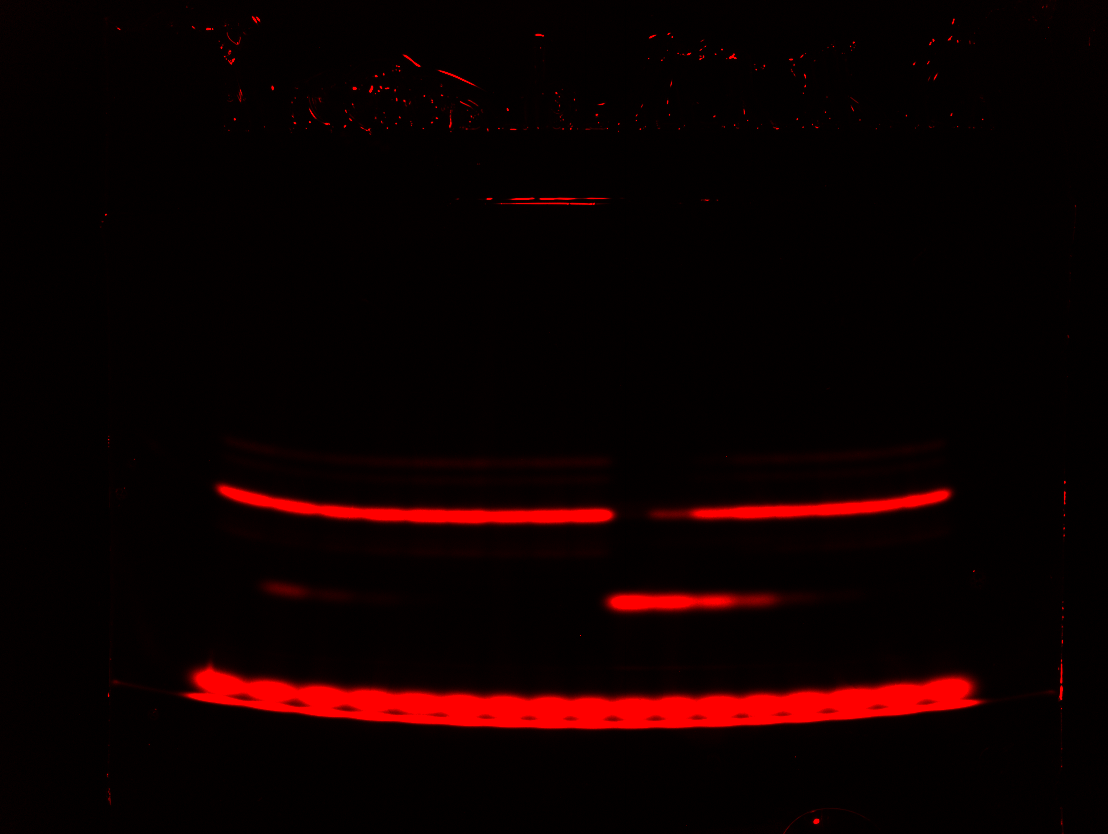

Supplement: Supplementary file 4 — Supplementary Data 1 [file 42003_2022_3444_MOESM4_ESM.zip › Unprocessed data/S Fig. 3/S Fig4B.png]

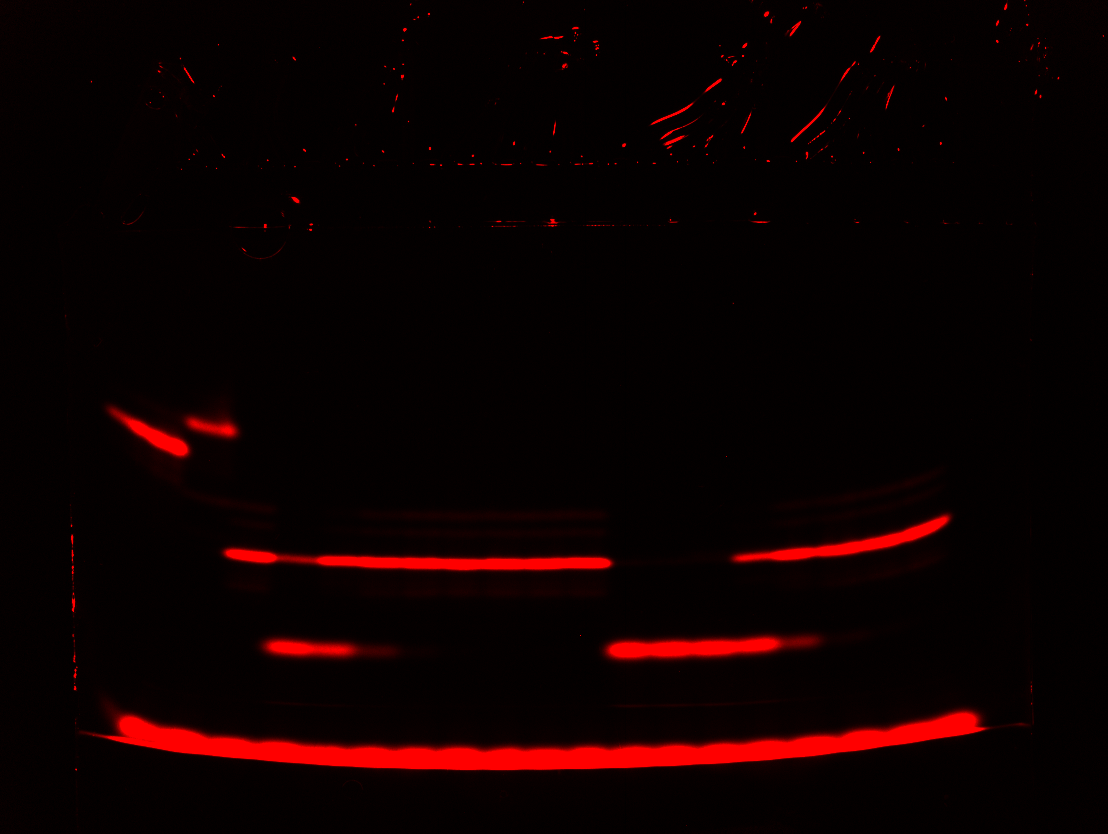

Supplement: Supplementary file 4 — Supplementary Data 1 [file 42003_2022_3444_MOESM4_ESM.zip › Unprocessed data/S Fig. 3/S Fig4C.png]

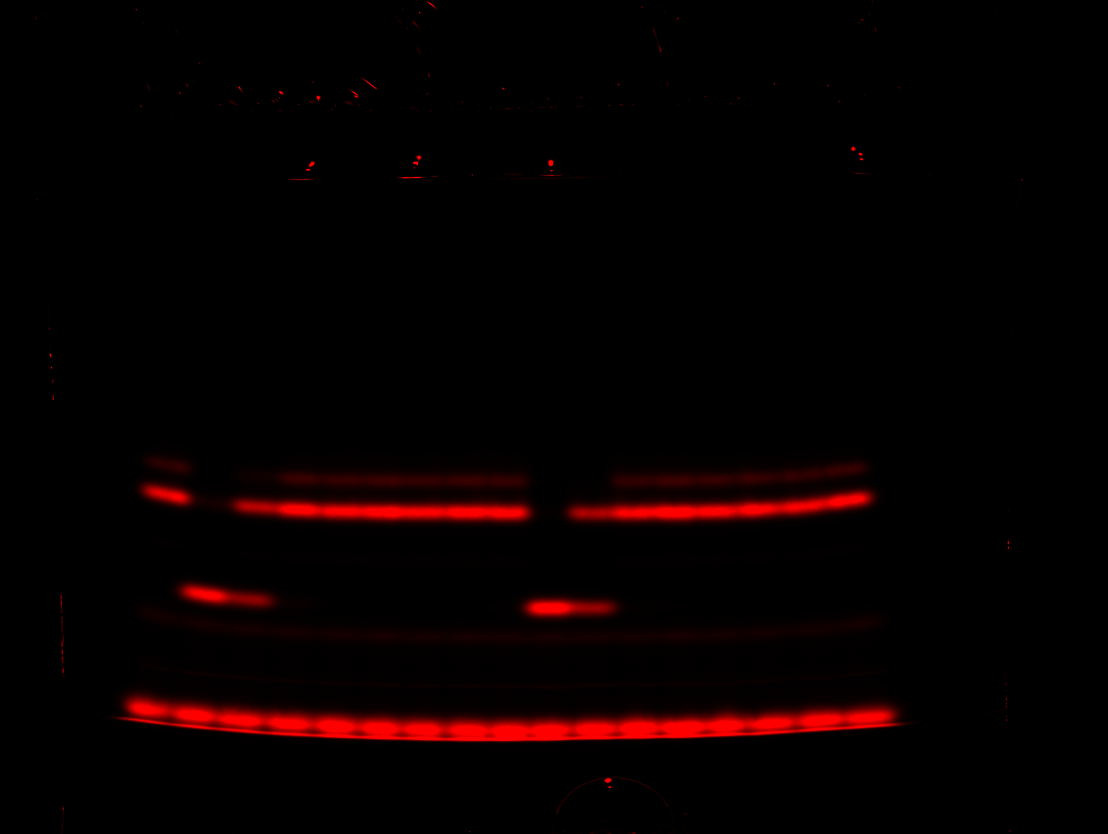

Supplement: Supplementary file 4 — Supplementary Data 1 [file 42003_2022_3444_MOESM4_ESM.zip › Unprocessed data/S Fig. 4/S Fig5A.png]

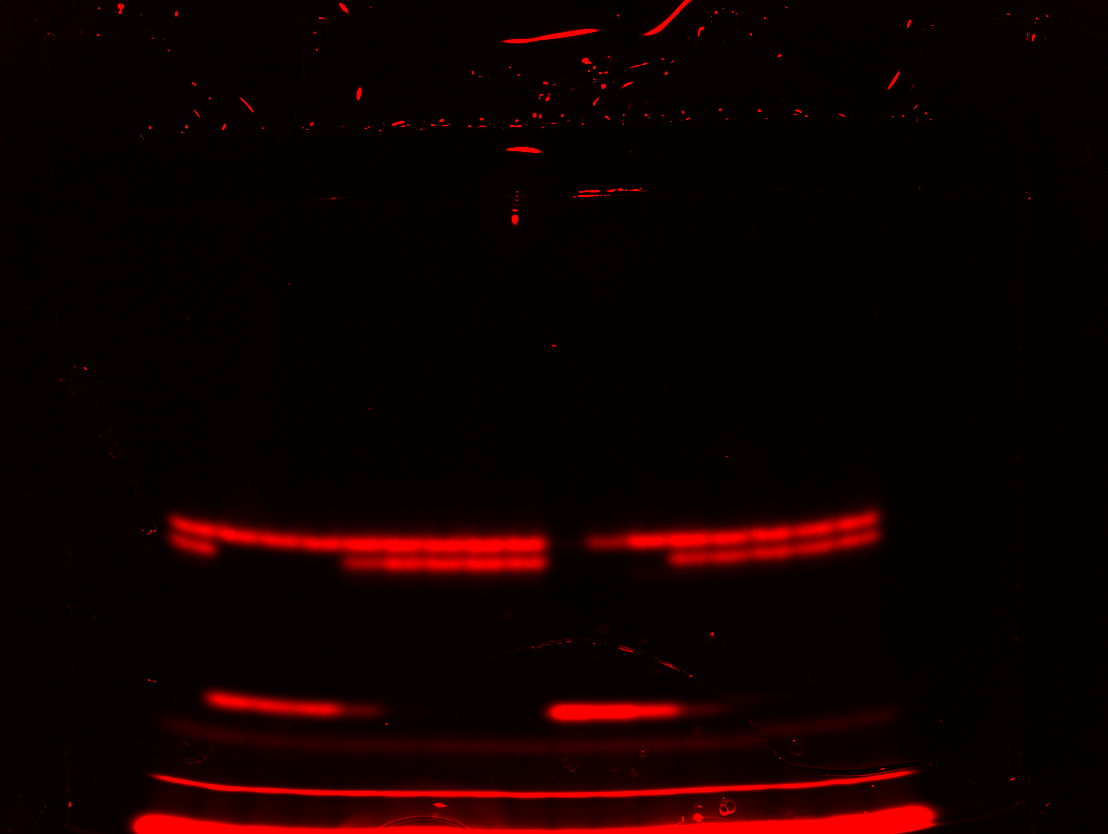

Supplement: Supplementary file 4 — Supplementary Data 1 [file 42003_2022_3444_MOESM4_ESM.zip › Unprocessed data/S Fig. 4/S Fig5B.png]

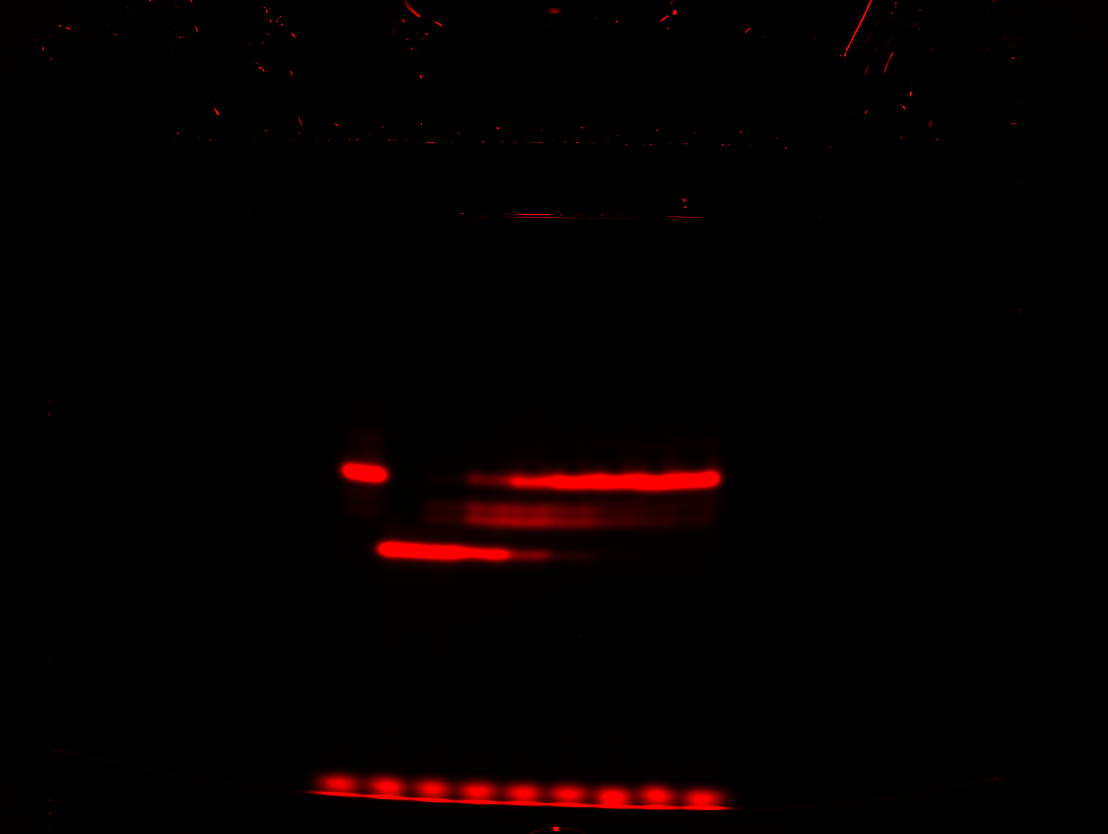

Supplement: Supplementary file 4 — Supplementary Data 1 [file 42003_2022_3444_MOESM4_ESM.zip › Unprocessed data/S Fig. 5/S Fig 6A1.png]

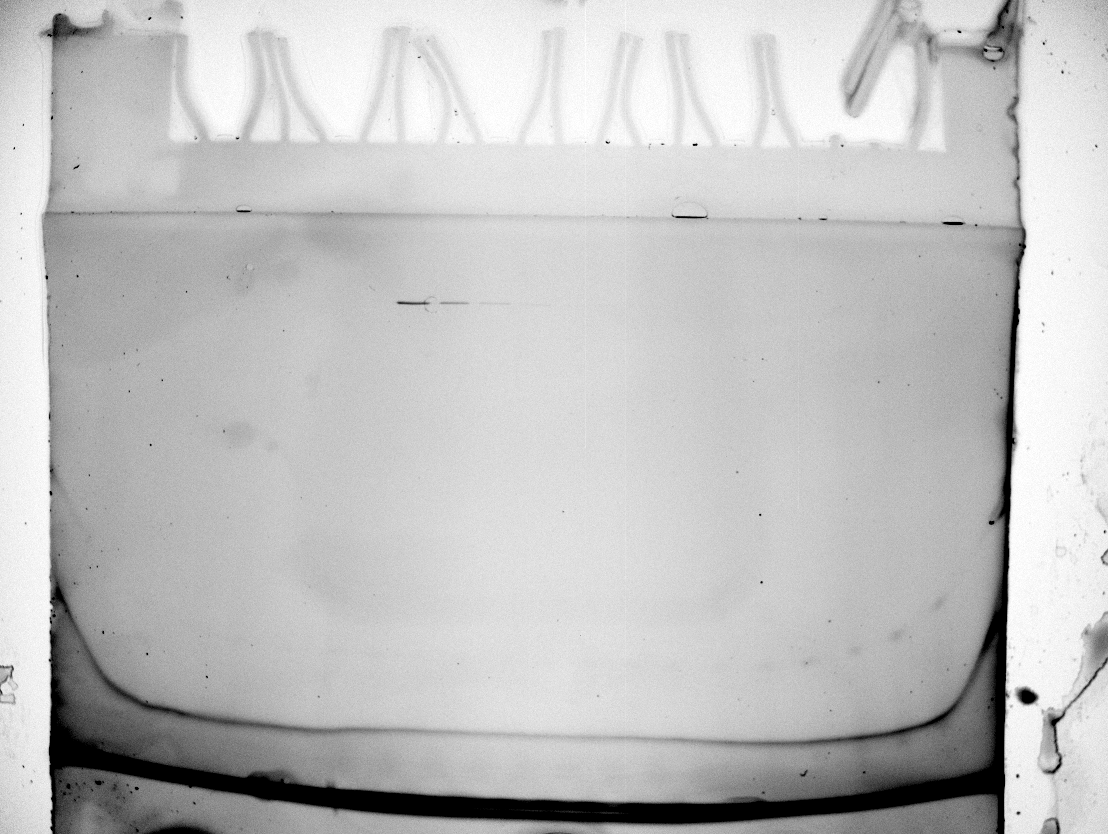

Supplement: Supplementary file 4 — Supplementary Data 1 [file 42003_2022_3444_MOESM4_ESM.zip › Unprocessed data/S Fig. 5/S Fig 6A2.png]

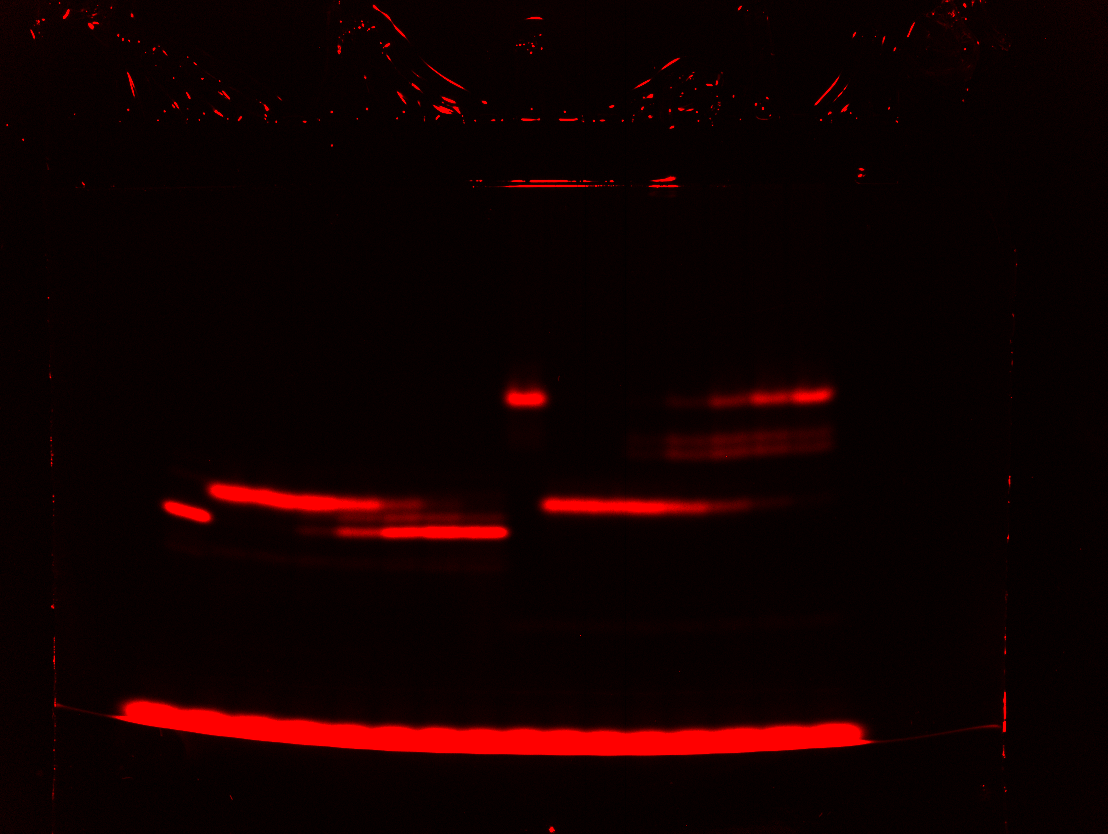

Supplement: Supplementary file 4 — Supplementary Data 1 [file 42003_2022_3444_MOESM4_ESM.zip › Unprocessed data/S Fig. 5/S Fig 6B1.png]

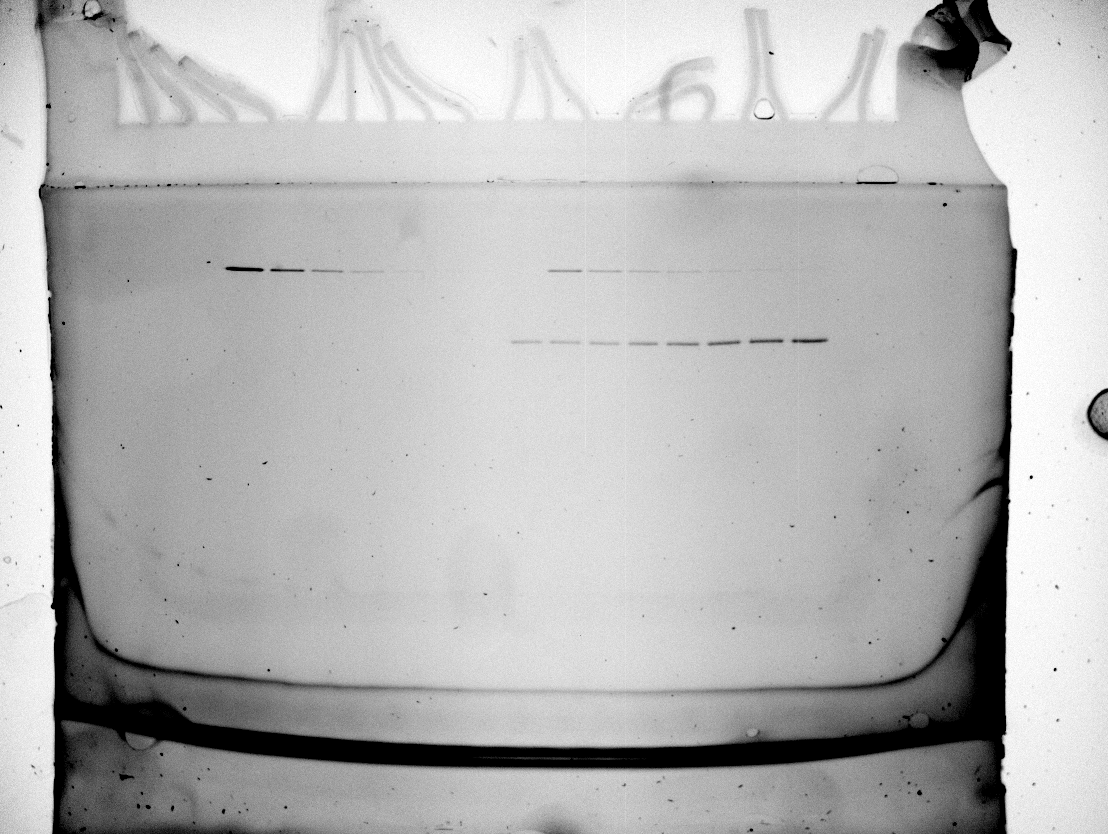

Supplement: Supplementary file 4 — Supplementary Data 1 [file 42003_2022_3444_MOESM4_ESM.zip › Unprocessed data/S Fig. 5/S Fig 6B2.png]
